# The influence of climate change on the potential distribution of *Ageratum conyzoides* in China

**DOI:** 10.1002/ece3.11513

**Published:** 2024-10-27

**Authors:** Yuan Wang, Yonggang Yang, Minggang Zhang

**Affiliations:** ^1^ Institute of Loess Plateau Shanxi University Taiyuan China; ^2^ School of Environmental and Resource Sciences Shanxi University Taiyuan China

**Keywords:** *Ageratum conyzoides*, biological invasion, climate change, climate niche, potential distribution

## Abstract

*Ageratum conyzoides* L., an invasive plant originating from South America, is characterized by rapid growth and strong ecological adaptability, posing a threat to China's ecosystems, agricultural industry, and biodiversity. In this study, we optimized the MaxEnt model using the ENMeval package and constructed an ensemble model using the Biomod2 package based on global geospatial distribution data of *A. conyzoides* and considering climate, soil, and topography factors. We simulated the potential suitable distribution of *A. conyzoides* in China at present and in the future (2041–2060, 2061–2080). Through multivariate environment similarity surface and most dissimilar variable analysis, we identified the main environmental variables influencing the distribution of *A. conyzoides*. Additionally, niche analysis elucidated temporal and spatial variations in *A. conyzoides*' climate niche. Our results demonstrate that the ensemble model, constructed from the top seven single models, outperforms the individual models in predicting the suitable habitat of *A. conyzoides*. The ensemble model achieved the true skill statistic (TSS) of 0.833 and the area under the subject curve (AUC) of 0.971, indicative of outstanding predictive performance. Presently, the suitable habitat of *A. conyzoides* in China primarily exists in the region between 18° and 28° N, covering approximately 1.47 million km^2^. The temperature annual range, precipitation of the wettest month, and mean temperature of the coldest quarter were identified as the primary environmental variables influencing its distribution, while soil and elevation variables had minor roles. Under future climate conditions, the suitable habitat of *A. conyzoides* is expected to expand northeastward, with the centroid of its habitat shifting northward as the climate warms. The migration speed of *A. conyzoides* is projected to increase with the degree of warming. Furthermore, the climate niche of *A. conyzoides* will undergo certain changes and may face both niche expansion and a decrease in niche overlap under different climate conditions.

## Graphical abstract

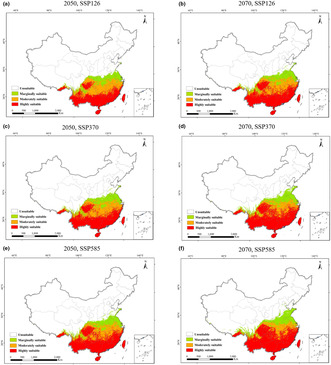

## 1 INTRODUCTION

Biological invasion poses a significant threat to biodiversity, referring to the process in which nonnative species enter and establish themselves in new ecosystems, causing harm to native species and impacting the native economy and environment (Reaser et al., 2020). These invasive species are characterized by their adaptability, high reproductive rates, and lack of natural predators, allowing them to rapidly expand and negatively influence local biodiversity, ecosystem stability, and economic development (Sakai et al., 2001; Wang et al., 2018; Wittenberg & Cock, 2001). Given the accelerated globalization and increased human activities, biological invasion has emerged as a critical aspect of global environmental issues (Ju et al., 2012; Mack et al., 2000). Therefore, in the context of climate change, it is essential to proactively manage and control potential invasive species in their suitable habitats to maintain ecological balance, preserve biodiversity, and promote sustainable development.

Species distribution models (SDMs) are valuable tools based on ecological niche theory that leverage environmental data and species distribution information to predict and explain the spatial distribution and ecological niche of species (Dormann et al., 2013; Liu et al., 2019). In the field of ecology and conservation biology, the rapid advancement of SDMs has made them essential for studying species distribution and understanding their environmental relationships (Beale & Lennon, 2012; Wiens et al., 2009). Species distribution is influenced by a multitude of biological, geographical, and environmental factors. Traditional survey methods for studying species distribution are often time‐consuming, costly, and limited to specific survey points. Conversely, SDMs utilize publicly available environmental data and existing species distribution records to develop mathematical and statistical models that can predict the potential distribution range of species in past or future. The use of SDMs is very important for the prevention and detection of invasive species (Chhogyel et al., 2021; Goldsmit et al., 2018; Liang, Tran, et al., 2018). Among SDMs, MaxEnt (Maximum Entropy) models are widely applied and have demonstrated good performance in numerous studies (Elith et al., 2011; Holder et al., 2020; Phillips et al., 2006; Phillips & Dudík, 2008). However, it is important to acknowledge that individual models have their own strengths and limitations, and when predicting species distribution ranges, results often display differences and high uncertainty (Segurado & Araújo, 2004). To address such limitations, researchers are increasingly from relying solely on a single model to employing an integrated approach that combines multiple models (Liu et al., 2011). This integration of well‐performing individual models effectively reduces model uncertainty (Araújo & New, 2007). Biomod2 is a widely used integrated model in recent years (Thuiller et al., 2009).

*Ageratum conyzoides* L., is a member of the Asteraceae family and is native to Central and North America, particularly Mexico. It possesses a rapid growth rate and wide ecological adaptability, resulting in its expansion in introduced areas. It is currently distributed widely in tropical and subtropical regions (Okunade, 2002). *A. conyzoides* exhibits strong ecological adaptability and exerts a significant inhibitory effect on other plants, placing it in a dominant position in interspecific competition (Luo et al., 2018). Its invasion poses a threat to local ecosystems, agricultural industries, and biodiversity (Kong, 2010). For instance, *A. conyzoides* inhibits and damages economic crops in farmlands, earning it the status of an important weed and causing economic losses in agricultural production (Devi & Khwairakpam, 2020; Kohli et al., 2006). The invasive plant also negatively impacts soil characteristics and species composition. *A. conyzoides* compete for soil nutrients and space, disrupting the structure and functioning of soil ecosystems and having adverse effects on soil quality (Maturi et al., 2024). Furthermore, the invasion of *A. conyzoides* has the potential to disrupt the balance and stability of local ecosystems (Kohli et al., 2006).

In the 1920s, *A. conyzoides* began its invasion of China, starting with the southern coastal regions, and by the 1930s, it had reached the southwestern border, particularly in southern Yunnan. Subsequently, it spread further inland, reaching provinces such as Hubei, Sichuan, Chongqing, Jiangxi, and Anhui (Wang, 2006). Presently, *A. conyzoides* primarily thrives in the Yangtze River Basin and southern regions (Zhong et al., 2016). Designated as an invasive weed (Ma, 2013), the rate of its expansion and the damage it causes suggest that *A. conyzoides* may continue to spread northward, potentially invading the entire Yangtze River basin (Hao & Qiang, 2005).

While some studies have investigated the physiological characteristics (Kaur et al., 2023), seed germination (Chen et al., 2018; Du et al., 2019), and interspecific competition (Chen et al., 2023) of *A. conyzoides*, there has been limited attention to the change of spatial distribution pattern of *A. conyzoides* post‐invasion in China. How will its spatial distribution pattern change in the future in China? What environmental factors drive its distribution, and how will its climatic niche change over time? To delve into these questions, we utilize collected distribution data of *A. conyzoides* and consider a range of environmental variables including temperature, precipitation, terrain, and soil. Through species distribution modeling and ecological niche analysis, we aim to shed light on several key aspects: (1) Determining the potential distribution range of *A. conyzoides* in China and identifying its primary limiting factors in the current period. (2) Assessing the impact of climate change on the potential distribution range of *A. conyzoides*. (3) Evaluating under different future climate scenarios, which environmental variables will have the greatest influence, positive or negative, on *A. conyzoides* within its suitable habitat. (4) Investigating how the geographical center of *A. conyzoides* distribution in China will shift from the present to the future. (5) Exploring the effects of climate change on *A. conyzoides*'s climatic niche. The findings of this study will serve as valuable references for the effective utilization and control of *A. conyzoides*.

## 2 MATERIALS AND METHODS

### 2.1 Species occurrence data

We collected global geographic distribution information of *A. conyzoides* from various reliable sources, such as the Chinese Virtual Herbarium (CVH, http://www.cvh.ac.cn/), the China National Specimen Information Infrastructure (NSII, http://www.nsii.org.cn/2017/home.php), and the Global Biodiversity Information Facility (GBIF, *Ageratum conyzoides* L. (gbif.org)). Initially, we eliminated distribution points with inaccurate or duplicate latitude and longitude coordinates, resulting in a total of 18,091 distribution points. To avoid overfitting caused by uneven distribution, we employed the ENMtool tool to filter densely distributed points (Warren et al., 2010). This filtering process randomly retained one distribution point in each 2.5′ × 2.5′ grid cell (covering an approximate area of 5 × 5 km), resulting in a final dataset containing 8596 distribution points (Figure S1).

### 2.2 Environmental variables

For this study, we selected three main environmental variables: climate, topography, and soil. All climate variables were obtained from the WorldClim database version 2.1 (https://www.worldclim.org), which provides a comprehensive dataset of 19 climate variables at a resolution of 2.5′. Topography variables, including elevation, slope, and aspect. The elevation data was also derived from the WorldClim database (https://www.worldclim.org), and slope and aspect data were obtained by processing the elevation data using the Surface Analyst Tool in ArcGIS software. Soil data, on the other hand, was obtained from the Harmonized World Soil Database (HWSD). The base maps used in this study were sourced from the Ministry of Natural Resources of the People's Republic of China (http://www.mnr.gov.cn/), with the map number GS (2019) 1822.

In terms of climate data, we used an ensemble of general circulation models (GCMs): ACCESS‐CM2, BCC‐CSM2‐MR, CMCC‐ESM2, IPSL‐CM6A‐LR, and MIROC6 (Fick & Hijmans, 2017; Wen et al., 2024). We used both current climate data covering the period of 1970–2000 and future climate data for the periods of 2041–2060 and 2061–2080. These predictions were based on the latest integrated scenarios of shared socioeconomic pathways (SSP) from the well‐regarded Coupled Model Intercomparison Project Phase 6 (CMIP6). We specifically focused on three representative scenarios: SSP1‐26 (low emission scenario), SSP3‐70 (medium emission scenario), and SSP5‐85 (high emission scenario). Each scenario represents a different range of future CO_2_ emissions, from low to high. All the climate data we selected have a resolution of 2.5′.

To address the issue of high collinearity among environmental variables and reduce overfitting (Guisan & Thuiller, 2005), we conducted a rigorous selection process in this study. A Pearson correlation analysis was performed among all 39 environmental variables. Variables with a correlation coefficient exceeding 0.75 were evaluated based on their importance in the model experiments, and only the variable with higher importance was retained (Table S1). Additionally, to improve computational efficiency, variables with an importance lower than 5% in the model experiments were removed. This selection process resulted in the inclusion of six climate variables, two topographic variables, and three soil variables (Table 2).

### 2.3 Model establishment and evaluation

In this study, we employed a combination of the Biomod2 and ENMeval packages for modeling. Initially, the ENMeval package was used to optimize the MaxEnt model by adjusting the feature combinations (FC) and regularization multipliers (RM) in order to obtain the optimal parameter combination (Muscarella et al., 2014). The MaxEnt model offers five feature combinations: linear (L), quadratic (Q), product (P), threshold (T), and hinge (H), with the default parameter setting as RM = 1 and FC = LQHPT. To optimize the model, RM was iteratively set between 0.5 and 4, incrementing by 0.5 in each run. The feature combinations were individually tested as L, LQ, H, LQH, LQHP, and LQHPT, resulting in a total of 48 parameter combinations. The model complexity and goodness of fit were evaluated using the delta.AICc (Akaike minimum information criterion; Phillips et al., 2006), while the model's overfitting was measured by AUC.diff (the difference between the mean of the training area under the subject curve (AUC) and the testing AUC) and OR10 (10% test omission rate; Muscarella et al., 2014).

After optimizing the MaxEnt model, we utilized the Biomod2 package to model the distribution of *A. conyzoides* using ten different models, namely, the generalized linear model (GLM), generalized boosted regression model (GBM), generalized additive model (GAM), classification and regression tree model (CTA), artificial neural network (ANN), multivariate adaptive regression splines (MARS), surface range envelogram (SRE), flexible discriminant analysis (FDA), random forest (RF), and maximum entropy value model (MaxEnt). With the exception of the MaxEnt model, which was optimized using the ENMeval package, the default parameters in Biomod2 were used for the remaining nine models. During the modeling process, 75% of the 8596 distribution points of *A. conyzoides* were randomly selected as the training dataset, while the remaining 25% constituted the testing dataset (Araujo et al., 2005). We chose the “random” method to generate 30,000 pseudo‐absences points and repeated it three times. The modeling process was repeated ten times.

To evaluate the accuracy of our models, we utilized two metrics: the area under the subject curve (AUC) and the true skill statistic (TSS). Both measurements range from 0 to 1, with values closer to 1 indicating better model performance. AUC values ranging from 0.8 to 1 and TSS values ranging from 0.8 to 1 indicate high model reliability (Allouche et al., 2006; Eskildsen et al., 2013).

### 2.4 Data analysis and processing

After the modeling is completed, the resulting data was then classified into four suitability levels using the reclassification function in ArcGIS 10.7 software. These levels were defined as unsuitable (0–0.3), marginally suitable (0.3–0.5), moderately suitable (0.5–0.7), and highly suitable (0.7–1; Wang et al., 2023). We evaluated the changes in the total suitable area by comparing the current and future predicted areas, calculating increase and decrease areas, as well as increase and decrease rates to assess changes in habitat suitability. We used the “Mean Center” tool in ArcGIS to calculate the centroids of *A. conyzoides* suitability habitat under different climate backgrounds. Based on the coordinates of these centroids, we determined the spatial movement direction and distance of the centroids, describing the overall trend of *A. conyzoides*'s future distribution (Jia et al., 2023).

### 2.5 Multivariate environment similarity surface (MESS) and most dissimilar (MoD) variable analysis

We utilized the current climate variables of *A. conyzoides* as the reference layer in our study to examine the effects of climate anomalies under future climate conditions and identify their key drivers. This involved employing a multivariate environmental similarity surface and analyzing the most dissimilar variables. The multivariate environmental similarity surface evaluates the resemblance between the climatic conditions at specific locations within a defined time period and the reference layer. It generates an overall similarity score by considering the similarity scores of each environmental factor, thereby quantifying the extent of climatic similarity between a location and the reference layer. Negative value results indicate climatic anomalies that exceed the range of corresponding values in the reference layer. Conversely, a value of 100 or higher indicates normal climatic patterns (Elith et al., 2010). The “most dissimilar variable” refers to the variable that displays the greatest deviation on the multivariate environmental similarity surface. It represents the key factor contributing to climatic anomalies at a specific point (Elith et al., 2010). Analyzing these most dissimilar variables enables the identification of key climate factors driving potential changes in suitable zones under different climate scenarios. This analysis was accomplished using the “density.tools.Novel” tool within the MaxEnt software (Zhang et al., 2020).

### 2.6 Climate niche change

We employed the Ecospat software package to perform climate niche analysis, using principal component analysis (PCA) to identify the key factors driving the differentiation of *A. conyzoides*'s ecological niche. We selected the distribution points and 1‐degree buffer distance as the background point selection area for the current climate and then utilized the suitable area predicted by ensemble models as the background point selection area for different future climatic scenarios (Gao, 2023). By randomly allocating current and future distribution points of *A. conyzoides* across various climate backgrounds, we quantified the niche overlap under different climatic conditions and assessed the stability of overlapping regions using tests for niche equivalency and similarity (Broennimann et al., 2012; Warren et al., 2008). The ecological niche parameter, Schoener's D, was employed to indicate the extent of niche overlap (Schoener, 1968), and we evaluated the similarity of species’ ecological niches by comparing the observed and simulated D values for both their resemblance and statistical significance. Last, we employed niche overlap to illustrate the changes in *A. conyzoides*'s ecological niche over time (Di Cola et al., 2017).

## 3 RESULTS

### 3.1 Model accuracy evaluation

**FIGURE 1:**
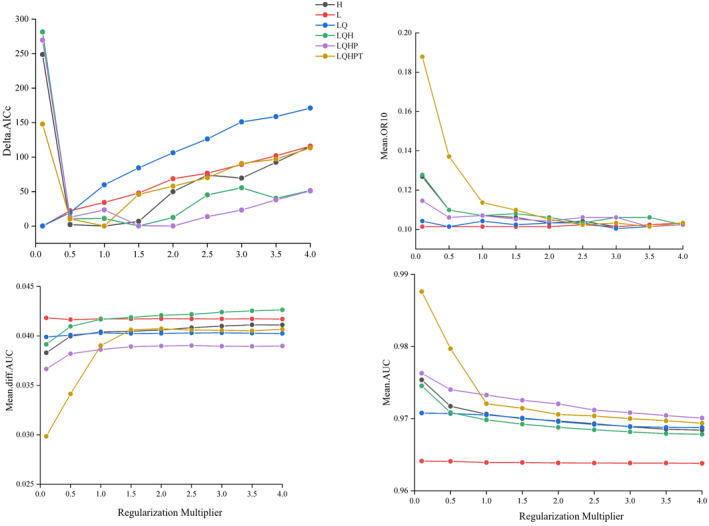
Performances for the MaxEnt model delta.AICc, mean diff. AUC, mean.OR10 and mean.AUC (‘mean AUC’ represents the performance on the test dataset).

Initially, we employed the ENMeval package to optimize various combinations of feature classes (FC) and regularization multipliers (RM). After thorough evaluation, we determined the optimal parameter configuration to be RM = 2 and FC = L, Q, H, and P, yielding a delta AICc value of 0 (Figure 1). Subsequent comparison revealed that the optimized MaxEnt model, with feature classes LQHP and regularization multiplier 2, exhibited significantly lower mean diff.AUC and mean OR10 values compared to the default parameter model (Figure 1). These findings indicate that the optimized MaxEnt model effectively addresses overfitting and complexity issues, resulting in enhanced predictive capability for determining the suitable habitat of *A. conyzoides* (Muscarella et al., 2014).

Next, we examined models that exhibited AUC values greater than 0.9 and TSS values exceeding 0.75. From our analysis, we identified seven models, including the Generalized Linear Model (GLM), Generalized Boosted Model (GBM), Classification Trees Analysis (CTA), Multiple Adaptive Regression Splines (MARS), Flexible Discriminant Analysis (FDA), Random Forest (RF), and MaxEnt (Table 1). To further enhance the accuracy of our predictions, we integrated these seven models using a weighted average approach, the ensemble model yielded a TSS of 0.833 and an AUC of 0.971 (Table 1).

**TABLE 1:** Comparison of true skill statistic (TSS) and area under the subject curve (AUC) values between single model and ensemble model.

Models	AUC	TSS
CTA	0.935 ± 0.004	0.815 ± 0.007
FDA	0.951 ± 0.002	0.782 ± 0.002
MARS	0.953 ± 0.002	0.799 ± 0.005
GBM	0.960 ± 0.002	0.815 ± 0.005
GLM	0.950 ± 0.002	0.789 ± 0.003
MAXENT	0.970 ± 0.002	0.790 ± 0.005
RF	0.999 ± 0.001	0.990 ± 0.001
EM	0.971	0.833

### 3.2 Importance of environmental variables

Based on the results obtained from the ensemble model using Biomod2, the contribution of different environmental variables to the distribution of *A. conyzoides* is outlined in Table 2. It can be observed that the contribution values of climate variables, terrain variables, and soil variables were found to be 94.18%, 2.11%, and 3.71%, respectively. The contribution of climate variables is the highest, followed by soil variables and terrain variables (Table 2).

**TABLE 2:** Main environmental factors and their importance of *Ageratum conyzoides*.

Variable type	Environment variable	Description	Unit	Importance
Climatic variables	BIO3	Isothermality	–	0.011
	BIO7	Temperature annual range	°C	0.400
	BIO10	Mean temperature of warmest quarter	°C	0.069
	BIO11	Mean temperature of coldest quarter	°C	0.186
	BIO13	Precipitation of wettest month	mm	0.262
	BIO14	Precipitation of driest month	mm	0.014
Topographical variables	Slope	Slope	°	0.001
	Aspect	Aspect	°	0.021
Soil variables	T‐TEB	Topsoil exchangeable base	cmol/kg	0.017
	T‐SAND	Topsoil sand fraction	%weight	0.003
	T‐pH‐H_2_O	Topsoil pH (H_2_O)	log (H^+^)	0.018

**FIGURE 2:**
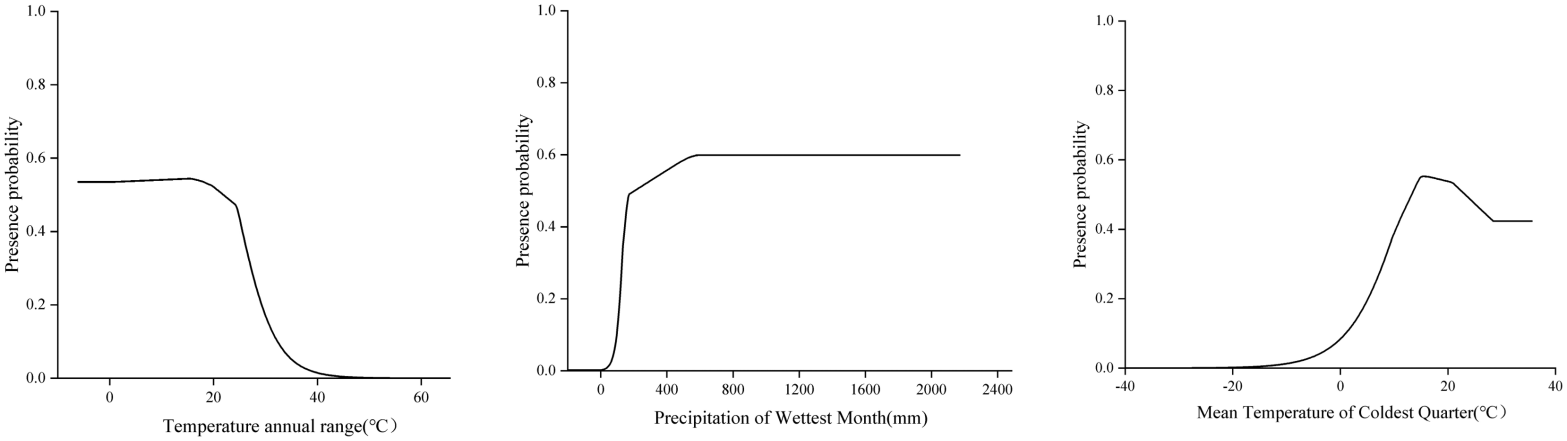
Response curves of major environmental factors.

To delve deeper into the individual factor analysis, the environmental factors exerting the greatest impact on the geographic distribution of *A. conyzoides*, along with their corresponding importance, are as follows: temperature annual range (bio7, 0.40), precipitation of wettest month (bio13, 0.27), mean temperature of coldest quarter (bio11, 0.19), and mean temperature of warmest quarter (bio10, 0.07). These four environmental factors collectively contribute over 90% to the modeling process, with the highest proportion (84.8%) attributed to the importance of the temperature annual range (bio7), precipitation of the wettest month (bio13), and mean temperature of the coldest quarter (bio11; Table 2). Hence, it can be inferred that the habitat of *A. conyzoides* is predominantly influenced by temperature and precipitation, particularly by the temperature annual range and the precipitation of the wettest month. On the other hand, both terrain and soil variables contribute less than 5%, and the impact of topography and soil on the distribution of *A. conyzoides* is relatively minor. According to the main environmental response curve (Figure 2), the optimal growth conditions for *A. conyzoides* are temperature annual range of −6 to 21°C, precipitation of the wettest month ranging from 500 to 2000 mm, and mean temperature of the coldest quarter between 13 and 23°C.

### 3.3 Potential distribution of *A. conyzoides* from the current to the future

#### 3.3.1 Present suitable distribution

**FIGURE 3:**
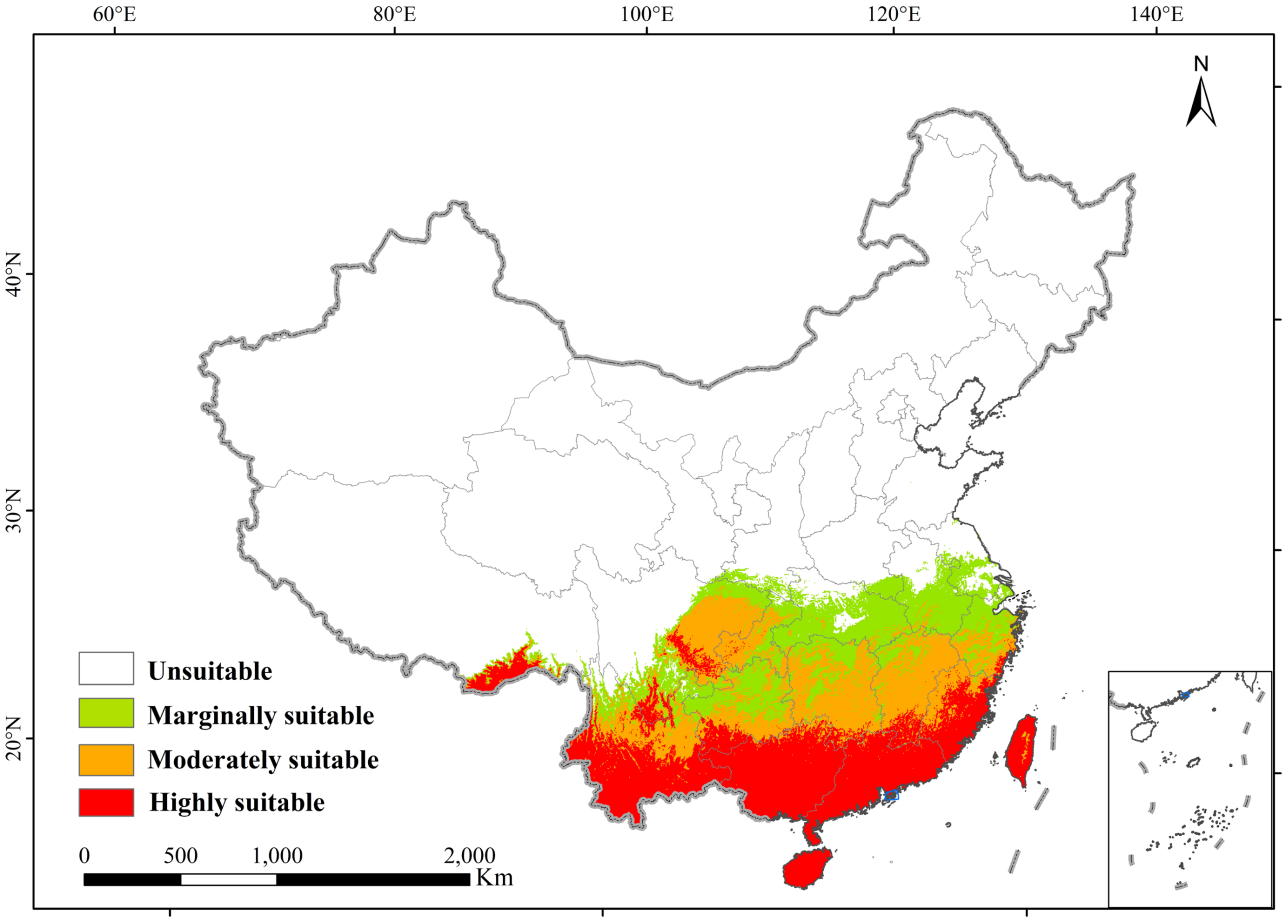
The potential distribution of *Ageratum conyzoides* in the current climate. The green, orange and red represent the marginally suitable, moderately suitable, and highly suitable areas, respectively.

Based on the findings presented in Figure 3, it can be observed that the current suitable habitat for *A. conyzoides* primarily exists in the lower latitude regions of China. This habitat spans a latitude range of 18°–28° N and a longitude range of 83°–130° E. The total area occupied by this region is estimated to be approximately 147.00 × 10^4^ km^2^, which accounts for 15.31% of the Chinese land area. Within the suitable habitat range, the high suitability habitat is concentrated in provinces such as Yunnan, Guangxi, Guangdong, Fujian, Hainan, and Taiwan, with sporadic occurrences in Sichuan and Tibet. The combined area of this high suitability habitat is roughly 56.21 × 10^4^ km^2^, encompassing 38.24% of the overall suitable habitat area. Encircling the high suitability habitat is the moderate suitability habitat, which covers regions in Yunnan, Fujian, Guizhou, Hunan, Jiangxi, Chongqing, and Sichuan. This habitat has a total area of approximately 46.29 × 10^4^ km^2^, constituting 31.49% of the entire suitable habitat area. Lastly, surrounding the moderate suitability habitat is the marginally suitability habitat, which includes areas in Hunan, Sichuan, Guizhou, Hubei, Anhui, and Zhejiang. The total estimated area of this habitat is approximately 44.50 × 10^4^ km^2^, accounting for 30.27% of the overall suitable habitat area (Table 3).

**TABLE 3:** Potential suitable habitat of *Ageratum conyzoides* in different periods (×10^4^ km^2^).

Period	Marginally suitable	Moderately suitable	Highly suitable	Total area	Area change	Area change ratio (%)
Current	44.501	46.290	56.211	147.001	**	**
2050s, SSP1‐2.6	36.155	53.006	74.572	163.732	44.464	37.065
2070s, SSP1‐2.6	37.080	47.861	89.239	174.180	49.344	41.133
2050s, SSP3‐7.0	38.373	50.462	85.142	173.977	45.948	38.302
2070s, SSP3‐7.0	39.992	42.363	101.485	183.840	64.631	53.876
2050s, SSP5‐8.5	36.577	45.772	93.924	176.274	53.363	44.484
2070s, SSP5‐8.5	43.261	37.306	112.740	193.307	74.561	62.154

** Don't compare the current period.

#### 3.3.2 Future distribution and changes

**FIGURE 4:**
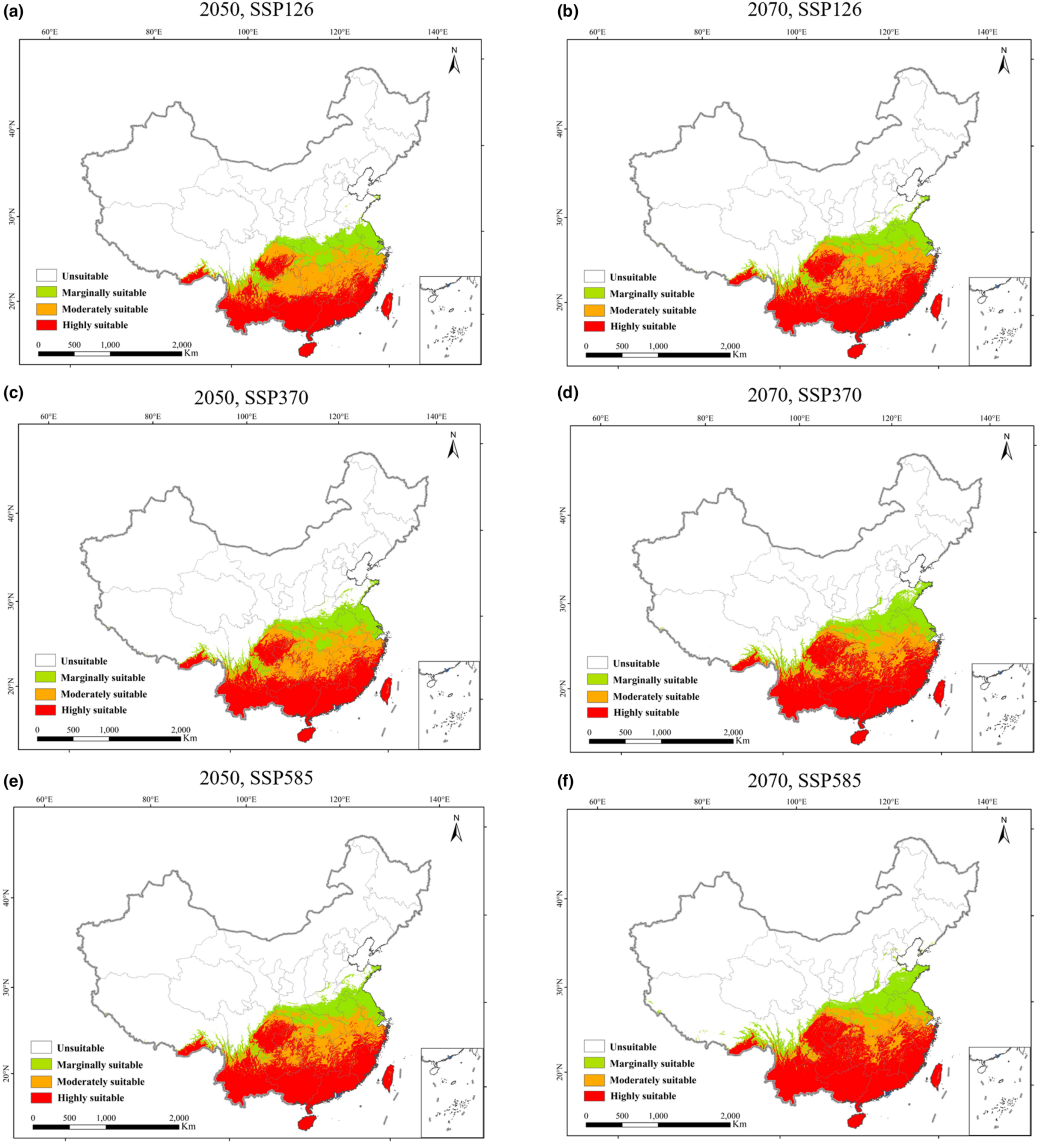
The potential distribution of *Ageratum conyzoides* in the future. The green, orange and red represent the marginally suitable, moderately suitable, and highly suitable areas, respectively. (a) under 2050s‐SSP1‐2.6, (b) under 2070s‐SSP1‐2.6, (c) under 2050s‐SSP3‐7.0, (d) under 2070s‐SSP3‐7.0, (e) 2050s‐SSP5‐8.5, and (f) under 2070s‐SSP5‐8.5.

**FIGURE 5:**
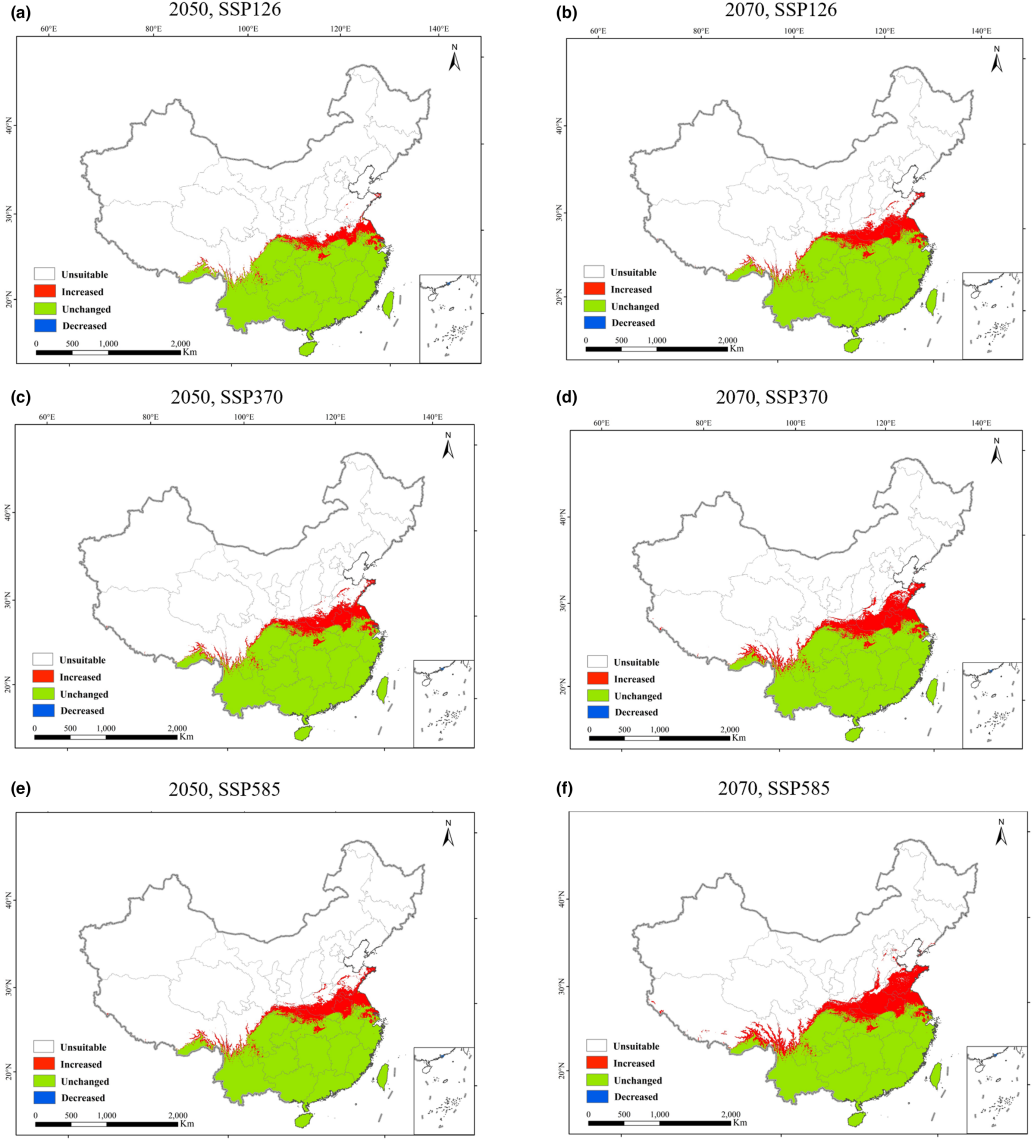
The expansion and contraction trend map of *Ageratum conyzoides* is inferred by overlaying the suitability distribution of the current and two future times (the 2050s and 2070s) based on three different emission scenarios (SSP1‐2.6, SSP3‐7.0, and SSP5‐8.5). Red, blue, green and white is depicting habitat increased, decreased, unchanged, and unsuitable, respectively. (a) The 2050s‐SSP1‐2.6, (b) the 2070s‐SSP1‐2.6, (c) the 2050s‐SSP3‐7.0, (d) the 2070s‐SSP3‐7.0, (e) the 2050s‐SSP5‐8.5, and (f) the 2070s‐SSP5‐8.5.

According to the projections in Figure 4, the future scenarios of climate change indicate a further expansion of suitable habitat for *A. conyzoides* toward north (Figure 4). This expansion is expected to mainly occur in the low latitude areas of China. Under the SSP126 scenario, the total suitable habitat for *A. conyzoides* is projected to increase to a range of 163.73 × 10^4^–174.18 × 10^4^ km^2^, representing a growth of 11.41%–18.49% compared to the current period. This expansion is primarily anticipated in provinces such as Shanxi, Henan, Jiangsu, Shanghai, Anhui, Shandong, and Hubei. In the SSP370 scenario, the total suitable habitat area is projected to expand even further, ranging from 173.98 × 10^4^–183.84 × 10^4^ km^2^, with an increase of 18.38%–25.06%. This expansion is expected to predominantly occur in Shanxi, Henan, Jiangsu, Shanghai, Anhui, Shandong, and Hubei. Similarly, under the SSP585 scenario, the total suitable habitat area is projected to expand to a range of 176.27 × 10^4^–193.31 × 10^4^ km^2^, representing an increase of 19.91%–31.53%. This expansion is anticipated in Xizang, Sichuan, Shanxi, Henan, Jiangsu, Shanghai, Anhui, Shandong, and Hubei. Importantly, it is worth noting that in all three scenarios, the expansion of suitable habitat for *A. conyzoides* primarily occurs in the northeastern part of its current distribution, and no reduction in its distribution occurs (Figure 5). Furthermore, as the emission concentration increases, the speed of habitat expansion for *A. conyzoides* also accelerates (Table 4). The SSP585 scenario shows the greatest increase in suitable habitat area and extent of expansion, followed by the SSP370 scenario, while the SSP126 scenario exhibits the least expansion.

**TABLE 4:** Differences in potentially suitable areas between two different time periods (2050s and 2070s) and three different circulation models (SSP1‐2.6, SSP3‐7.0, and SSP5‐8.5).

Period	Decrease (×10^4^ km^2^)	Increase (×10^4^ km^2^)	Unchanged (×10^4^ km^2^)	Decrease rate (%)	Increase rate (%)	Range change (%)
2050s, SSP1‐2.6	0	16.772	147.002	0	11.409	11.409
2070s, SSP1‐2.6	0	27.179	147.002	0	18.489	18.489
2050s, SSP3‐7.0	0	27.017	147.002	0	18.378	18.378
2070s, SSP3‐7.0	0	36.839	147.002	0	25.060	25.06
2050s, SSP5‐8.5	0	29.273	147.002	0	19.913	19.913
2070s, SSP5‐8.5	0	46.347	147.002	0	31.528	31.528

Comparing the marginally, moderate, and high suitability habitats across the future climate scenarios of SSP126, SSP370, and SSP585, it is evident that the moderate suitability habitat decreases in all three scenarios, with only a minor increase observed. The marginally suitability habitat is also decreasing in all three scenarios. Conversely, the high‐suitability habitats experience an increase across the scenarios (Table 3). The marginally suitability habitat contracts by a range of 1.24 × 10^4^–8.35 × 10^4^ km^2^, with a decrease rate of 2.79–18.75%. The moderate suitability habitat shows a change in area from −8.98 × 10^4^ km^2^ to 6.72 × 10^4^ km^2^, with a change rate of −19.41% to 14.51%. The high suitability habitat expands by an area ranging from 16.73 × 10^4^ to 46.31 × 10^4^ km^2^, with an increase rate of 29.77%–82.38% (Table 3). The high suitability habitat experiences the fastest growth, suggesting that the expansion of suitable habitat for *A. conyzoides* is primarily driven by the transformation of unsuitable areas, marginally and moderately suitable habitats in the current period, into high suitability habitats in the future. The warmer climate creates more favorable conditions for the survival and spread of *A. conyzoides*.

### 3.4 Multivariate environment similarity surface (MESS) and most dissimilar (MoD) variable analysis

**FIGURE 6:**
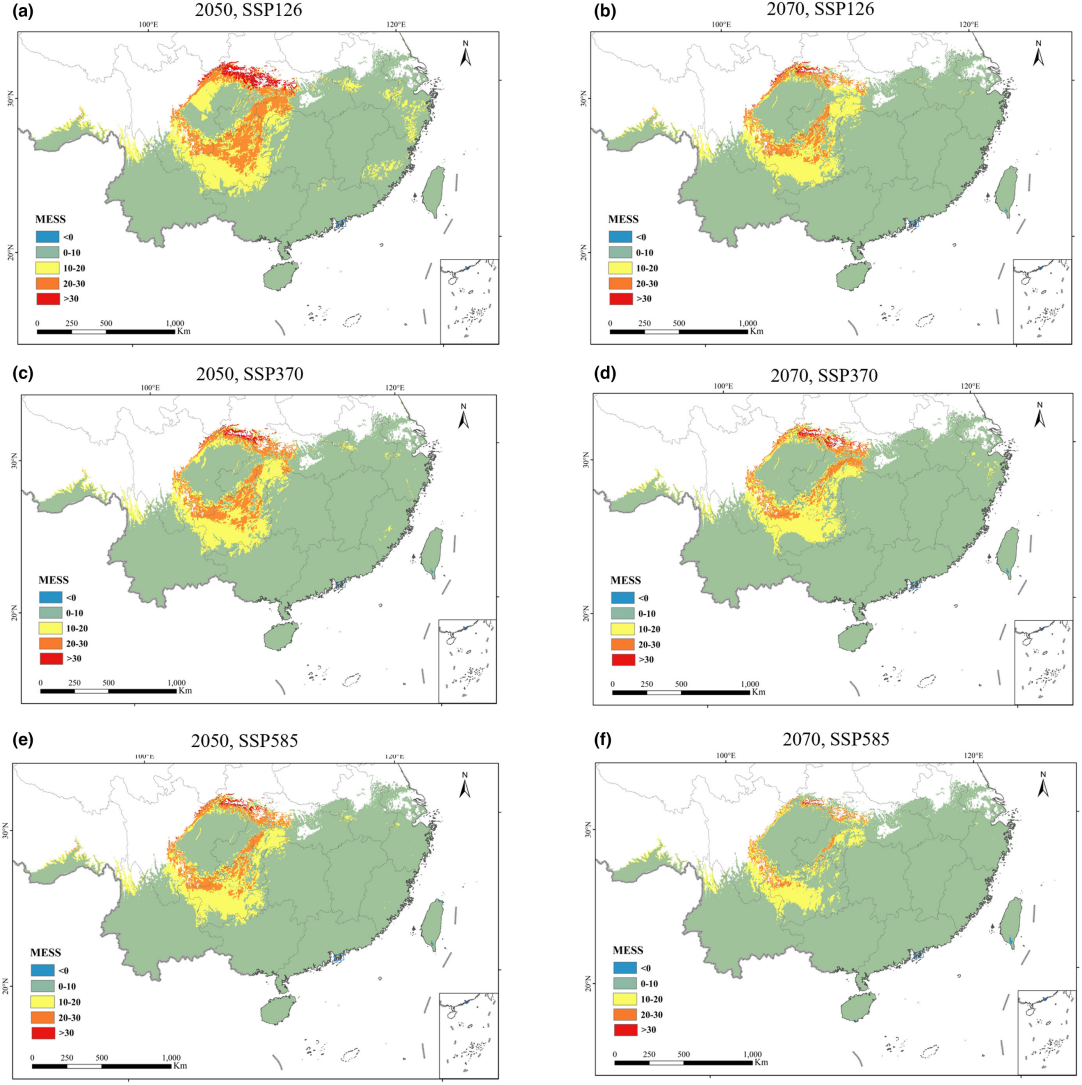
Multivariate environmental similarity surface (MESS) in the potential distribution area of *Ageratum conyzoides* under different climate scenarios. (a) The 2050s‐SSP1‐2.6, (b) the 2070s‐SSP1‐2.6, (c) the 2050s‐SSP3‐7.0, (d) the 2070s‐SSP3‐7.0, (e) the 2050s‐SSP5‐8.5, and (f) the 2070s‐SSP5‐8.5.

**FIGURE 7:**
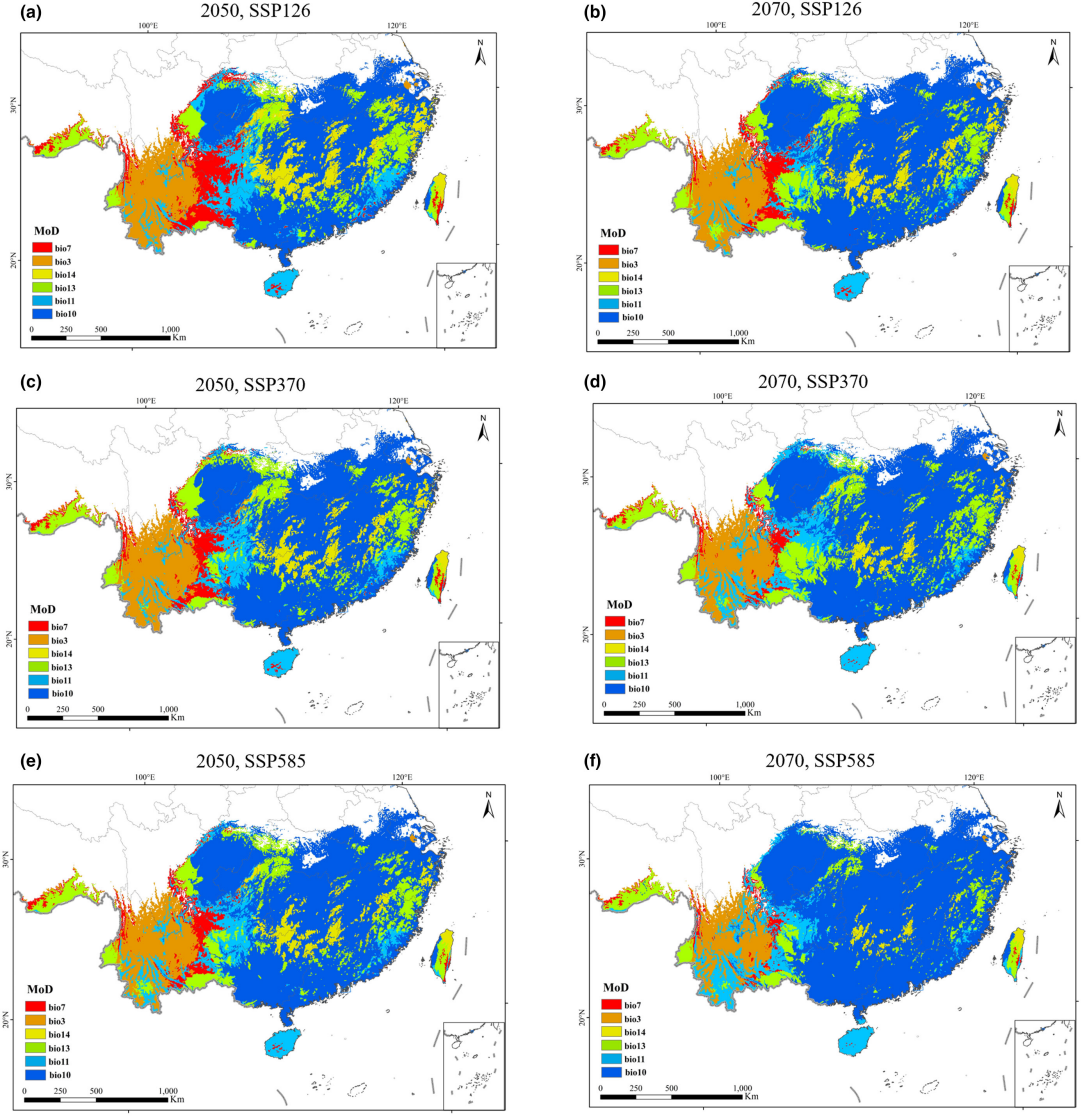
The most dissimilar variable (MoD) in the potential distribution area of *Ageratum conyzoides* under different climate scenarios. (a) The 2050s‐SSP1‐2.6, (b) the 2070s‐SSP1‐2.6, (c) the 2050s‐SSP3‐7.0, (d) the 2070s‐SSP3‐7.0, (e) the 2050s‐SSP5‐8.5, and (f) the 2070s‐SSP5‐8.5.

Based on the results of the Multi‐Environment Similarity Surface (MESS) and Most Dissimilar (MoD) variables analysis, the differences in the present suitable distribution areas of *A. conyzoides* under six different future emission conditions are mostly within the range of 0–10. This indicates a relatively low variation among the distribution points of *A. conyzoides*, with an average MESS value ranging from 2.49 to 6.32 for its present suitable distribution. The SSP585 scenario in the 2070s exhibits the highest climate anomaly level, with an average MESS value of 2.49. It is closely followed by the SSP370 scenario. On the other hand, the climate anomaly level is lowest in the 2050s under the SSP126 conditions, with an average MESS value of 6.32. Geographically, certain regions in the northern part of *A. conyzoides*'s present distribution area show MESS values exceeding 10, indicating higher climate anomalies in the southern regions compared to the northern regions (Figure 6). The most dissimilar variables contributing to the environmental changes include the mean temperature of the warmest quarter (bio10), isothermality (bio3), and precipitation of the wettest month (bio13; Figure 7).

### 3.5 Centroid migration

**FIGURE 8:**
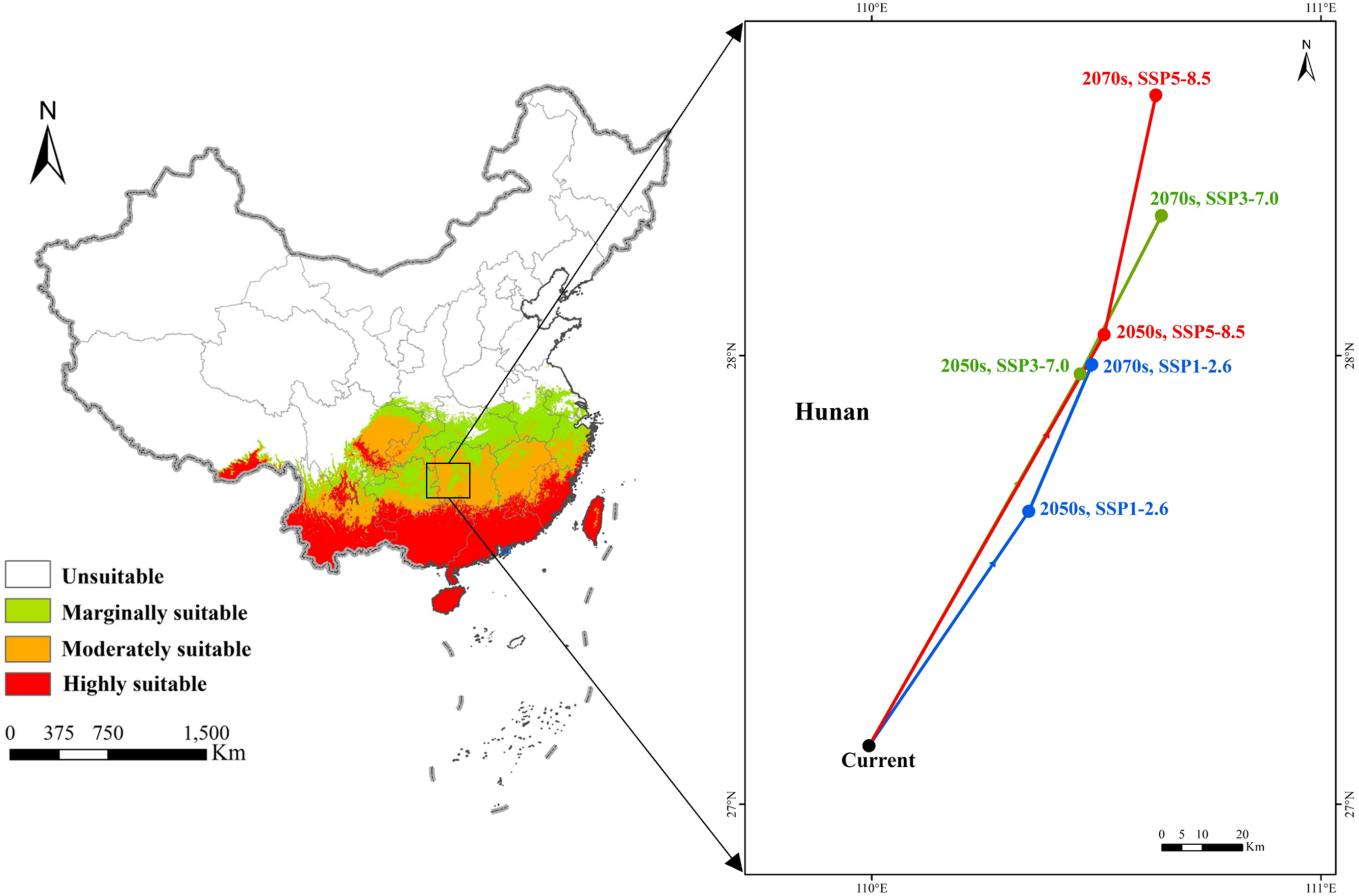
Centroid shifts under current and future climate scenarios for *Ageratum conyzoides*.

The centroid of a habitat reflects its central position and can be used to infer the overall migration trend. In our study, we calculated the centroids of the suitability areas for *A. conyzoides* under different periods and climate scenarios. Interestingly, the centroids consistently show a northward movement trend, which is in line with the observed change in the species' distribution area (Figure 8). Currently, the centroid of *A. conyzoides* distribution is located in Hunan province (109°99′36″ E, 27°13′02″ N), while the centroids under all three future scenarios are situated in Hunan province.

Under the SSP126 climate scenario, the centroid initially shifts northeastward by 105.5 km (110°34′94″ E, 27°65′30″ N) and then continues to move in the same direction, covering a total distance of 38.5 km (110°48′99″ E, 27°98′02″ N). In the SSP370 scenario, the centroid moves northeastward by 105.8 km (110°46′40″ E, 27°95′90″ N), and then continues to move in the same direction, covering an additional distance of 49.7 km (110°64′50″ E, 28°31′22″ N). In the SSP585 scenario, the centroid initially shifts northeastward by 128.9 km (110°51′73″ E, 28°04′68″ N) and then continues to move in the same direction, covering a further distance of 110.7 km (110°63′26″ E, 28°58′09″ N). Among the three scenarios, *A. conyzoides* exhibits the longest migration path under the SSP585 scenario, followed by the SSP370 scenario, while the SSP126 scenario demonstrates the shortest migration distance.

### 3.6 Analysis of climate niche change

According to PCA, the first two principal components (PC) explain 77.65%–80.03% of the variation in climate variables within the ecological niche of *A. conyzoides* (PC1: 39.13%–42.49%; PC2: 35.71%–37.75%; Figure S2). The mean temperature of the warmest quarter (bio10), isothermality (bio3), and temperature annual range (bio7) are identified as the main driving factors influencing the changes in the ecological niche of *A. conyzoides* related to climate.

The results of our climate niche analysis demonstrate that as the climate warms, there is a continuous decrease in the overlap between *A. conyzoides*'s niche under future climatic conditions and its current climate niche. Additionally, there is a slight expansion of the niche, indicating a relatively high level of stability (Table 5). In the SSP126 climate scenario, the overlap between *A. conyzoides*'s climate niche in the 2050s and its current niche is highest, reaching 0.769. Conversely, under the SSP585 climate scenario, the overlap between *A. conyzoides*'s climate niche in the 2070s and its current niche is lowest, at only 0.699 (Table 5).

**TABLE 5:** Climatic niche changes of *Ageratum conyzoides*.

Period	2050s, SSP1‐2.6	2070s, SSP1‐2.6	2050s, SSP3‐7.0	2070s, SSP3‐7.0	2050s, SSP5‐8.5	2070s, SSP5‐8.5
Niche stability (NS)	1.000	0.999	0.998	0.998	0.998	0.997
Niche expansion (NE)	0.000	0.001	0.002	0.002	0.002	0.003
Niche overlap	0.769	0.754	0.733	0.708	0.721	0.699

## 4 DISCUSSION

### 4.1 Optimization results and accuracy of the model

In our modeling approach, we used ENMtools to select distribution points and employed Pearson correlation analysis to eliminate highly correlated environmental variables. This helped to reduce overfitting and improve the robustness of our model. However, it is important to note that our study only focused on abiotic factors, neglecting many other important factors including land use, physiological traits, and anthropogenic factors (Laeseke et al., 2020; Zhu et al., 2017). This limitation may affect the accuracy of our future dispersal predictions for *A. conyzoides* under ideal conditions. Furthermore, sampling biases and imprecise measurements of environmental variables could introduce deviations in our predictions. These factors are what we will try to improve in the future.

To reduce uncertainty in the modeling process, we adopted a combined approach using the ENMeval and Biomod2 packages, this approach has been shown to effectively enhance the transferability of the MaxEnt model and reduce model complexity (Zhao et al., 2021). The evaluation metrics used, including delta.AICc, mean AUC.diff, and mean OR10, indicated that the ENMeval package successfully improved the performance of the MaxEnt model. By optimizing the MaxEnt model and combining it with other high‐performing models such as GLM, GBM, CTA, MARS, FDA, and RF, we created an ensemble model through a weighted average approach. The TSS and AUC values of the ensemble model were higher than those of any individual model, demonstrating that the ensemble approach improved the accuracy and reliability of our results.

### 4.2 Dominant climatic factor

Species‐environment relationships play a critical role in understanding the ecological requirements and spatial distribution of species. Our study reveals that the distribution of *A. conyzoides* is primarily influenced by temperature and precipitation, especially the temperature annual range (bio7), precipitation of the wettest month (bio13), and mean temperature of the coldest quarter (bio11; Table 2). Previous research has established that *A. conyzoides*, which originates from subtropical regions, is sensitive to cold temperatures and ceases growth when the temperature drops below 8°C (Liu et al., 2011). Our findings strongly support this notion. However, it's worth noting that despite its temperature sensitivity, *A. conyzoides* displays remarkable adaptability, with no strict requirements for altitude and soil conditions, as indicated by the relatively minor influence of altitude and soil factors in our study.

The significant adaptability and phenotypic plasticity of *A. conyzoides* have been widely documented. For instance, Chen et al. (2023) have demonstrated its ability to adapt to varying temperature, humidity, soil texture, and altitude conditions, which aligns with our findings. Moreover, Zhang et al. (2024) observed the seed viability of *A. conyzoides* under different soil temperature and moisture conditions, revealing its substantial tolerance to soil variations. Similarly, Du et al. (2019) studied the seed germination of *A. conyzoides* under different temperature, light, pH, and salinity conditions, uncovering its broad adaptive range. Furthermore, Xu et al. (2019) investigated the survival and reproduction of *A. conyzoides* under diverse environmental conditions, highlighting its adaptability to light and soil conditions, as well as its resilience to adverse environments, this evidence suggests that *A. conyzoides* can become one of the dominant weeds in farmland because of its strong ecological adaptability (Kohli et al., 2006).

### 4.3 The change of the suitable distribution pattern

After analyzing the potential distribution areas of *A. conyzoides* across three climate scenarios (SSP126, SSP370, and SSP585), it becomes apparent that the species exhibits a consistent trend of expansion. Notably, the rate of expansion accelerates with increasing degrees of climate warming, projecting a faster spread by 2070 compared to 2050. This phenomenon can be attributed to *A. conyzoides*' natural affinity towards subtropical regions and its adaptability to temperatures ranging between 15 and 30°C (Kaur et al., 2023). This adaptability enables the species to flourish in both higher altitude (temperate) and low‐lying (tropical) areas (Kaur et al., 2023). The warm and humid climate, abundant sunlight, and favorable geographical positioning of southern China create ideal conditions for the optimal growth and reproduction of *A. conyzoides*. Its formidable ecological adaptability and allelopathic effects confer upon it a competitive advantage, fueling rapid growth and expansion within the Yangtze River Basin and southern regions of China (Zhang et al., 2024). Furthermore, predictions from the Biomod2 model suggest that *A. conyzoides* still harbors significant potential for further expansion. By around 2050, it is anticipated to saturate ecological niches in subtropical and tropical regions before gradually encroaching into temperate areas. However, its expansion into temperate regions may encounter limitations imposed by climate factors, particularly the lowest temperature during the temperature annual range (bio7) and the mean temperature of the coldest quarter (bio11), as these exhibit substantial annual fluctuations. Although *A. conyzoides* demonstrates moderate tolerance to low temperatures, its growth rate could be impeded by sensitivity to such conditions.

Additionally, Figure 5 illustrates an eastward bias in the expansion of *A. conyzoides*. The species exhibits a faster spread towards provinces like Shandong and Henan compared to its westward progression along the Hengduan Mountains in the north. This variance can be attributed to the complex terrain and high altitudes of the Hengduan Mountains, which act as barriers to dispersion (Liang, Xu, et al., 2018). Moreover, the harsh and cold climate in high‐altitude regions presents additional obstacles to its westward expansion.

Our analysis of the multivariate environmental similarity surface and the most dissimilar variables reveal an interesting pattern: there is a greater extent of anomalies in the southern distribution area compared to the northern region. However, as climate warming progresses, this north–south difference is gradually diminishing. The level of environmental heterogeneity within most distribution areas ranges from 0 to 10. Furthermore, with increasing climate warming, the zones with climate anomalies are steadily shifting towards higher latitudes. We also have identified the mean temperature of the warmest quarter (bio10) and the isothermality (bio3) as the primary factors driving these changes. These variables play a crucial role in influencing the distribution of *A. conyzoides*. It is evident that higher levels of climate anomalies have a negative impact on the spread of *A. conyzoides*, underscoring the significance of temperature in shaping its distribution patterns. In particular, low temperatures emerge as the key limiting factors restraining the northward expansion of *A. conyzoides*.

In our analysis of the centroid displacement of *A. conyzoides* across different climate scenarios, a consistent trend emerges: the migration speed accelerates with increasing degrees of climate warming. Notably, this migration predominantly occurs towards the north. As climate warming affects subtropical and temperate regions of China, alterations in temperature and hydrological conditions render these areas more hospitable to the survival and expansion of *A. conyzoides*. These observations align with similar trends seen in other invasive plants within the Asteraceae family. For instance, *Mikania micrantha*, another invasive species, has shown an expansion of its suitable habitat and a significant northward shift under projected future climate conditions (Zeng et al., 2024). Likewise, *Ageratina adenophora*, also from the Asteraceae family, is gradually expanding its suitable habitat in China, primarily migrating eastward and northward in the future (Wei et al., 2022). Furthermore, studies have indicated that in the face of climate warming, various species adapt by migrating to higher altitudes or latitudes (Bentley et al., 2018; Chen et al., 2011; Suon et al., 2019). These findings provide additional evidence supporting the observed migration trend of *A. conyzoides*.

### 4.4 Analysis of climate niche change

In our analysis of the dynamic changes in the climate niche of *A. conyzoides* using the Ecospat package, we observe a gradual reduction in the degree of niche overlap as the climate warms. The mean temperature of the warmest quarter (bio10), isothermality (bio3), and temperature annual range (bio7) emerge as the primary factors influencing its ecological niche. Expanding on our prior findings, we posit that the differentiation of *A. conyzoides*' ecological niche likely stems from its expansion into new territories prompted by rising temperatures. This expansion enables it to occupy a broader ecological niche, elucidating the potential increase in the distribution area of *A. conyzoides* under various future climate scenarios, along with its ongoing movement towards the northeast.

The ornamental value of *A. conyzoides*, is increasingly recognized, leading to its gradual introduction and spread as a horticultural plant further northward. However, it is crucial to avoid indiscriminate cultivation solely for ornamental or medicinal purposes. Instead, there should be a focus on comprehensive research and practical utilization strategies. In the future, efforts regarding the utilization and control of *A. conyzoides* invasion should consider the following key points: Firstly, further investigation into the species' response to specific environmental variables include temperature and precipitation could provide a more nuanced understanding of its distribution dynamics. Secondly, assessing the efficacy of various management strategies to control the spread of *A. conyzoides*, such as biological control methods or targeted eradication efforts, especially in economically developed areas with favorable environmental conditions such as Guizhou, Hunan, Jiangxi, Zhejiang, Sichuan, and Chongqing, it would be invaluable for mitigating its impact on local ecosystems. Thirdly, given the projected expansion of *A. conyzoides*' suitable habitat under future climate scenarios, it is essential to monitor its spread and assess its impacts on native biodiversity. Long‐term ecological studies examining how *A. conyzoides* interacts with native flora and fauna, as well as its potential to alter ecosystem functioning, are warranted. Such research could provide insights into the broader ecological implications of its invasion and inform conservation efforts. Fourthly, incorporating socio‐economic factors into future studies would enhance our understanding of the broader context in which *A. conyzoides* invasion occurs. Investigating the socio‐economic drivers of its spread, as well as the socio‐economic impacts on local communities, can inform more holistic management strategies that consider both ecological and human dimensions.

By addressing these key research areas, future studies can contribute to a more comprehensive understanding of the ecological implications of *A. conyzoides* invasion and facilitate the development of effective management and conservation strategies.

## 5 CONCLUSIONS

Our study employed the Biomod2 software package to construct an ensemble model for predicting the future distribution of *A. conyzoides* under different climate scenarios. The current suitable habitat for *A. conyzoides* is primarily concentrated in the region between 18° and 28° N in China, with particular emphasis on Guangxi, Guangdong, Fujian, and Hainan provinces. Our findings indicate that temperature and precipitation are the major environmental factors influencing the distribution of *A. conyzoides*, with low temperatures being a limiting factor for its expansion. However, our research also suggests that *A. conyzoides* has the potential for further dispersion, especially in the context of global warming, its suitable habitat is projected to expand and progressively move towards higher latitudes in the future. Furthermore, as the climate warms, the ecological niche of *A. conyzoides* may expand and reduce overlap slightly.

The continuous expansion of *A. conyzoides* presents a worrisome trend for the local ecosystem. As an invasive plant, it poses a significant threat to the local ecosystem, agricultural industry, and biodiversity. It is crucial to implement effective management and control strategies urgently to combat the invasion of *A. conyzoides*. This includes strengthening monitoring and early warning mechanisms to promptly detect and report the spread of invasive species. Additionally, comprehensive research on the biology and ecology of *A. conyzoides* should be conducted to provide a scientific basis for its prevention and control. Promoting the application of biological control methods, such as introducing natural enemies, can help manage its population and mitigate its impact. Furthermore, raising public awareness and education about invasive species prevention and control is essential in enhancing understanding and consciousness in addressing this issue.

## AUTHOR CONTRIBUTIONS

**Yuan Wang:** Conceptualization (equal); data curation (equal); software (equal); visualization (equal); writing – original draft (equal). **Yonggang Yang:** Funding acquisition (equal); resources (equal); software (equal); writing – review and editing (equal). **Minggang Zhang:** Funding acquisition (equal); project administration (equal); resources (equal); supervision (equal).

## FUNDING INFORMATION

The Key Research and Development Program of Shanxi Province, Grant Number: 202304290000016; The National Natural Science Foundation of China, Grant Number: 31700465.

## CONFLICT OF INTEREST STATEMENT

The authors declare no conflict of interest.

## Supporting information


Data S1.

Data S2.

## Data Availability

The data that supports the findings of this study are available in Data S1 and S2.

## References

[ece311513-bib-0001] Allouche, O. , Tsoar, A. , & Kadmon, R. (2006). Assessing the accuracy of species distribution models: Prevalence, kappa and the true skill statistic (TSS). Journal of Applied Ecology, 43(6), 1223–1232. 10.1111/j.1365-2664.2006.01214.x

[ece311513-bib-0002] Araújo, M. B. , & New, M. (2007). Ensemble forecasting of species distributions. Trends in Ecology and Evolution, 22(1), 42–47. 10.1016/j.tree.2006.09.010 17011070

[ece311513-bib-0003] Araujo, M. B. , Pearson, R. G. , Thuiller, W. , & Erhard, M. (2005). Validation of species‐climate impact models under climate change. Global Change Biology, 11(9), 1504–1513. 10.1111/j.1365-2486.2005.01000.x

[ece311513-bib-0004] Beale, C. M. , & Lennon, J. J. (2012). Incorporating uncertainty in predictive species distribution modelling. Philosophical Transactions of the Royal Society of London. Series B, Biological Sciences, 367, 247–258. 10.1098/rstb.2011.0178 22144387 PMC3223803

[ece311513-bib-0005] Bentley, L. K. , Robertson, M. P. , & Barker, N. P. (2018). Range contraction to a higher elevation: The likely future of the montane vegetation in South Africa and Lesotho. Biodiversity and Conservation, 28, 131–153. 10.1007/s10531-018-1643-6

[ece311513-bib-0006] Broennimann, O. , Fitzpatrick, M. C. , Pearman, P. B. , Petitpierre, B. , Pellissier, L. , Yoccoz, N. G. , Thuiller, W. , Fortin, M. , Randin, C. , Zimmermann, N. E. , Graham, C. H. , & Guisan, A. (2012). Measuring ecological niche overlap from occurrence and spatial environmental. Global Ecology and Biogeography, 21, 481–497. 10.1111/j.1466-8238.2011.00698.x

[ece311513-bib-0007] Chen, I.‐C. , Hill, J. K. , Ohlemüller, R. , Roy, D. B. , & Thomas, C. D. (2011). Rapid range shifts of species associated with high levels of climate warming. Science, 333(6045), 1024–1026. 10.1126/science.1206432 21852500

[ece311513-bib-0008] Chen, S. , Ye, Z. , Ran, H. , Lan, X. , Zhou, P. , & He, Y. (2018). Effect of different environmental condition on biological characteristics of *Ageratum conyzoides*, an exotic invasion weed. Guizhou Agricultural Sciences, 46(9), 64–66.

[ece311513-bib-0009] Chen, X. , Zhang, Y. , Zhang, Y. , & Luo, Z. (2023). Response of phenotypic plasticity of invasive *Ageratum conyzoides* L. to interspecific competition. Plant Science Journal, 41(1), 37–43.

[ece311513-bib-0010] Chhogyel, N. , Kumar, L. , & Bajgai, Y. (2021). Invasion status and impacts of parthenium weed (*Parthenium hysterophorus*) in west‐central region of Bhutan. Biological Invasions, 23, 2763–2779. 10.1007/s10530-021-02534-3

[ece311513-bib-0011] Devi, C. , & Khwairakpam, M. (2020). Feasibility of vermicomposting for the management of terrestrial weed *Ageratum conyzoides* using earthworm species *Eisenia fetida* . Environmental Technology and Innovation, 18, 100696. 10.1016/j.eti.2020.100696

[ece311513-bib-0012] Di Cola, V. , Broennimann, O. , Petitpierre, B. , Breiner, F. T. , d'Amen, M. , Randin, C. , Engler, R. , Pottier, J. , Pio, D. , & Dubuis, A. (2017). ecospat: An R package to support spatial analyses and modeling of species niches and distributions. Ecography, 40, 774–787. 10.1111/ecog.02671

[ece311513-bib-0013] Dormann, C. F. , Elith, J. , Bacher, S. , Buchmann, C. , Carl, G. , Carré, G. , Marquéz, J. R. G. , Gruber, B. , Lafourcade, B. , & Leitão, P. J. (2013). Collinearity: A review of methods to deal with it and a simulation study evaluating their performance. Ecography, 36(1), 27–46. 10.1111/j.1600-0587.2012.07348.x

[ece311513-bib-0014] Du, L. , Li, R. , Dong, Y. , Huang, B. , Fu, Y. , & Tang, D. (2019). Seed germination and seedling emergence of *Ageratum conyzoides* in response to different environmental factors. Acta Ecologica Sinica, 39(15), 5662–5669.

[ece311513-bib-0015] Elith, J. , Kearney, M. , & Phillips, S. (2010). The art of modelling range‐shifting species. Methods in Ecology and Evolution, 1(4), 330–342. 10.1111/j.2041-210X.2010.00036.x

[ece311513-bib-0016] Elith, J. , Phillips, S. J. , Hastie, T. , Dudík, M. , Chee, Y. E. , & Yates, C. J. (2011). A statistical explanation of MaxEnt for ecologists. Diversity and Distributions, 17(1), 43–57. 10.1111/j.1472-4642.2010.00725.x

[ece311513-bib-0017] Eskildsen, A. , le Roux, P. C. , Heikkinen, R. K. , Høye, T. T. , Kissling, W. D. , Pöyry, J. , Wisz, M. S. , & Luoto, M. (2013). Testing species distribution models across space and time: High latitude butterflies and recent warming. Global Ecology and Biogeography, 22(12), 1293–1303. 10.1111/geb.12078

[ece311513-bib-0018] Fick, S. E. , & Hijmans, R. J. (2017). WorldClim 2: New 1‐km spatial resolution climate surfaces for global land areas. International Journal of Climatology, 37(12), 4302–4315.

[ece311513-bib-0019] Gao, M. (2023). Effects of environmental changes on the degradation of secondary Betula platyphylla forests in the north of the greater Khingan Mountains. Inner Mongolia Agricultural University.

[ece311513-bib-0020] Goldsmit, J. , Archambault, P. , Chust, G. , Villarino, E. , Liu, G. , Lukovich, J. V. , Barber, D. G. , & Howland, K. L. (2018). Projecting present and future habitat suitability of ship‐mediated aquatic invasive species in the Canadian Arctic. Biological Invasions, 20, 501–517. 10.1007/s10530-017-1553-7

[ece311513-bib-0021] Guisan, A. , & Thuiller, W. (2005). Predicting species distribution: Offering more than simple habitat models. Ecology Letters, 8, 993–1009. 10.1111/j.1461-0248.2005.00792.x 34517687

[ece311513-bib-0022] Hao, J. , & Qiang, S. (2005). New invasive weed–*Ageratum conyzoides* L. Weed Science, 4, 54–58.

[ece311513-bib-0023] Holder, A. M. , Markarian, A. , Doyle, J. M. , & Olson, J. R. (2020). Predicting geographic distributions of fishes in remote stream networks using maximum entropy modeling and landscape characterizations. Ecological Modelling, 433, 109231. 10.1016/j.ecolmodel.2020.109231

[ece311513-bib-0024] Jia, T. , Qi, Y. , Zhao, H. , Xian, X. , Li, J. , Huang, H. , Yu, W. , & Liu, W. (2023). Estimation of climate‐induced increased risk of *Centaurea solstitialis* L. invasion in China: An integrated study based on biomod2. Frontiers in Ecology and Evolution, 11, 1113474. 10.3389/fevo.2023.1113474

[ece311513-bib-0025] Ju, R. T. , Li, H. , Shih, C. J. , & Li, B. (2012). Progress of biological invasions research in China over the last decade. Biodiversity Science, 20(5), 581–611. 10.3724/SP.J.1003.2012.31148

[ece311513-bib-0026] Kaur, A. , Kaur, S. , Singh, H. P. , Datta, A. , Chauhan, B. S. , Ullah, H. , Kohli, R. K. , & Batish, D. R. (2023). Ecology, biology, environmental impacts, and Management of an Agro‐Environmental Weed *Ageratum conyzoides* . Plants, 12, 2329. 10.3390/plants12122329 37375954 PMC10301300

[ece311513-bib-0027] Kohli, R. K. , Batish, D. R. , Singh, H. P. , & Dogra, K. S. (2006). Status, invasiveness and environmental threats of three tropical American invasive weeds (*Parthenium hysterophorus* L, *Ageratum conyzoides* L., *Lantana camara* L.) in India. Biological Invasions, 8, 1501–1510. 10.1007/s10530-005-5842-1

[ece311513-bib-0028] Kong, C. H. (2010). Ecological pest management and control by using allelopathic weeds (*Ageratym conzoides*, *Ambrosia trifida*, and *Lantan camara*) and their allelochemicals in China. Weed Biology and Management, 10(2), 73–80. 10.1111/j.1445-6664.2010.00373.x

[ece311513-bib-0029] Laeseke, P. , Martínez, B. , Mansilla, A. , & Bischof, K. (2020). Future range dynamics of the red alga *Capreolia implexa* in native and invaded regions: Contrasting predictions from species distribution models versus physiological knowledge. Biological Invasions, 22, 1339–1352. 10.1007/s10530-019-02186-4

[ece311513-bib-0030] Liang, Q. , Xu, X. , Mao, K. , Wang, M. , Wang, K. , Xi, Z. , & Liu, J. (2018). Shifts in plant distributions in response to climate warming in a biodiversity hotspot, the Hengduan Mountains. Journal of Biogeography, 45(6), 1334–1344. 10.1111/jbi.13229

[ece311513-bib-0031] Liang, W. , Tran, L. , Washington‐Allen, R. , Wiggins, G. , Stewart, S. , Vogt, J. , & Grant, J. (2018). Predicting the potential invasion of kudzu bug, *Megacopta cribraria* (Heteroptera: Plataspidae), in north and South America and determining its climatic preference. Biological Invasions, 20, 2899–2913. 10.1007/s10530-018-1743-y

[ece311513-bib-0033] Liu, H. , Yang, J. , & Yang, H. (2011). Propagation and cultivation management techniques of *Ageratum conyzoides* L. Agriculture Engineering Technology (Greenhouse Horticultore), 8, 46.

[ece311513-bib-0034] Liu, X. , Yuan, Q. , & Ni, J. (2019). Research advances in modelling plant species distribution in China. Acta Phytoecologica Sinica, 43(4), 273–283.

[ece311513-bib-0036] Luo, Z. , Chen, X. , Xia, G. , & Chen, B. (2018). Extrinsic environmental factors, not resident diversity itself, lead to invasion of *Ageratum conyzoides* L. in diverse communities. Ecological Research, 33(6), 1245–1253. 10.1007/s11284-018-1637-6

[ece311513-bib-0037] Ma, J. S. (2013). The checklist of the invasive plants (p. 177). Higher Education Press.

[ece311513-bib-0038] Mack, R. N. , Simberloff, D. , Mark Lonsdale, W. , Evans, H. , Clout, M. , & Bazzaz, F. A. (2000). Biotic invasions: Causes, epidemiology, global consequences, and control. Ecological Applications, 10, 689–710. 10.1002/ece3.10711

[ece311513-bib-0039] Maturi, K. C. , Haq, I. , & Kalamdhad, A. S. (2024). Insights into the bioconversion of *Ageratum conyzoides* into a nutrient‐rich compost and its toxicity assessment: Nutritional and quality assessment through instrumental analysis. Biomass Conversion and Biorefinery, 14, 3879–3895. 10.1007/s13399-022-02532-y

[ece311513-bib-0040] Muscarella, R. , Galante, P. J. , Soley‐Guardia, M. , Boria, R. A. , Kass, J. M. , Uriarte, M. , & Anderson, R. P. (2014). ENMeval: An R package for conducting spatially independent evaluations and estimating optimal model complexity for Maxent ecological niche models. Methods in Ecology and Evolution, 5(11), 1198–1205. 10.1111/2041-210X.12261

[ece311513-bib-0041] Okunade, A. L. (2002). *Ageratum conyzoides* L. Fitoterapia, 73(1), 1–16. 10.1016/S0367-326X(01)00364-1 11864757

[ece311513-bib-0042] Phillips, S. J. , Anderson, R. P. , & Schapire, R. E. (2006). Maximum entropy modeling of species geographic distributions. Ecological Modelling, 190(3–4), 231–259. 10.1016/j.ecolmodel.2005.03.026

[ece311513-bib-0043] Phillips, S. J. , & Dudík, M. (2008). Modeling of species distributions with Maxent: New extensions and a comprehensive evaluation. Ecography, 31(2), 161–175. 10.1111/j.0906-7590.2008.5203.x

[ece311513-bib-0044] Reaser, J. K. , Burgiel, S. W. , Kirkey, J. , Brantley, K. A. , Veatch, S. D. , & Burgos‐Rodríguez, J. (2020). The early detection of and rapid response (EDRR) to invasive species: A conceptual framework and federal capacities assessment. Biological Invasions, 22(1), 1–19. 10.1007/s10530-019-02156-w

[ece311513-bib-0045] Sakai, A. K. , Allendorf, F. W. , Holt, J. S. , Lodge, M. , Molofsky, J. , With, K. A. , Cabin, R. J. , Cohen, J. E. , Norman, C. , Mccauley, D. E. , Neil, P. O. , Parker, M. , Thompson, J. N. , & Weller, S. G. (2001). The population biology of invasive species. Annual Review of Ecology and Systematics, 32, 305–332. 10.1146/annurev.ecolsys.32.081501.114037

[ece311513-bib-0046] Schoener, T. W. (1968). Anolis lizards of Bimini: Resource partitioning in a complex fauna. Ecology, 49, 704–726. 10.2307/1935534

[ece311513-bib-0047] Segurado, P. , & Araújo, M. B. (2004). An evaluation of methods for modeling species distributions. Journal of Biogeography, 31, 1555–1568. 10.1111/j.1365-2699.2004.01076.x

[ece311513-bib-0048] Suon, S. , Li, Y. , Porn, L. , & Javed, T. (2019). Spatiotemporal analysis of soil moisture drought over China during 2008‐2016. Journal of Water Resource and Protection, 11(6), 700–712. 10.4236/jwarp.2019.116041

[ece311513-bib-0049] Thuiller, W. , Lafourcade, B. , Engler, R. , & Araújo, M. B. (2009). BIOMOD – A platform for ensemble forecasting of species distributions. Ecography, 32, 369–373. 10.1111/j.1600-0587.2008.05742.x

[ece311513-bib-0050] Wang, L. , Liu, J. , Liu, J. , Wei, H. , Fang, Y. , Wang, D. , Chen, R. , & Gu, W. (2023). Revealing the long‐term trend of the global‐scale *Ginkgo biloba* distribution and the impact of future climate change based on ensemble modeling. Biodiversity and Conservation, 32, 2077–2100. 10.1007/s10531-023-02593-z

[ece311513-bib-0051] Wang, R. (2006). Historical reconstruction of invasion and expansion and potential spread of some threatening invasive alien species in China. Institute of Botany, Chinese Academy of Sciences.

[ece311513-bib-0052] Wang, W. J. , He, H. S. , Thompson, F. R. , Spetich, M. A. , & Fraser, J. S. (2018). Effects of species biological traits and environmental heterogeneity on simulated tree species distribution shifts under climate change. Science of the Total Environment, 634, 1214–1221. 10.1016/j.scitotenv.2018.03.353 29710627

[ece311513-bib-0053] Warren, D. L. , Glor, R. E. , & Turelli, M. (2008). Environmental niche equivalency versus conservatism: Quantitative approaches to niche evolution. Evolution, 62(11), 2868–2883. 10.1111/j.1558-5646.2008.00482.x 18752605

[ece311513-bib-0054] Warren, D. L. , Glor, R. E. , & Turelli, M. (2010). ENMTools: A toolbox for comparative studies of environmental niche models. Ecography, 33, 607–611. 10.1111/j.1600-0587.2009.06142.x

[ece311513-bib-0055] Wei, B. , Liu, L. , Gu, C. , Yu, H. , Zhang, Y. , Zhang, B. , Cui, B. , Gong, D. , & Tu, Y. (2022). The climate niche is stable and the distribution area of *Ageratina adenophora* is predicted to expand in China. Biodiversity Science, 30(8), 88–99.

[ece311513-bib-0056] Wen, X. , Fang, G. , Chai, S. , He, C. , Sun, S. , Zhao, G. , & Lin, X. (2024). Can ecological niche models be used to accurately predict the distribution of invasive insects? A case study of *Hyphantria cunea* in China. Ecology and Evolution, 14, e11159. 10.1002/ece3.11159 38487749 PMC10940052

[ece311513-bib-0057] Wiens, J. A. , Stralberg, D. , Jongsomjit, D. , Howell, C. A. , & Snyder, M. A. (2009). Niches, models, and climate change: Assessing the assumptions and uncertainties. Proceedings of the National Academy of Sciences of the United States of America, 106(Suppl 2), 19729–19736. 10.1073/pnas.0901639106 19822750 PMC2780938

[ece311513-bib-0058] Wittenberg, R. , & Cock, M. J. W. (2001). Invasive alien species: A toolkit of best prevention and management practices. CAB International.

[ece311513-bib-0059] Xu, W. , Zheng, S. , & Yu, Q. (2019). The effects of environmental factors on morphology, survival and fecundity of *Ageratum conyzoides* . Journal of Lishui University, 41(5), 34–40.

[ece311513-bib-0060] Zeng, X. , Yu, S. , Tian, M. , & Zhang, N. (2024). Suitable growth areas of the invasive plant *Mikania micrantha* in Guizhou and its trends. Journal of Biosafety, 33(2), 169–176.

[ece311513-bib-0061] Zhang, Y. B. , Liu, Y. L. , Zhang, X. L. , Qin, H. , Wang, G. Y. , & Wang, W. (2020). Impacts of climate change on the suitable habitats and spatial migration of *Xanthoceras sorbifolia* . China Environmental Science, 40(10), 4597–4606.

[ece311513-bib-0062] Zhang, Y. , Ouyang, A. , Wang, F. , Jia, C. , Yang, F. , & Tang, D. (2024). Seed viability of *Ageratum conyzoides* on different soil environments. Journal of Nanjing Agricultural University, 47(2), 284–291.

[ece311513-bib-0063] Zhao, G. , Cui, X. , Sun, J. , Li, T. , Wang, Q. , Ye, X. , & Fan, B. (2021). Analysis of the distribution pattern of Chinese *Ziziphus jujuba* under climate change based on optimized biomod2 and Maxent models. Ecological Indicators, 132, 108256. 10.1016/j.ecolind.2021.108256

[ece311513-bib-0064] Zhong, J. , Zhou, H. , Liu, K. , Yuan, C. , Li, X. , & Liu, W. (2016). Comparative study on seed biological characteristics of three invasive plants of Compositae, *Bidens bipinnata*, *Cirsium japonicum* and *Eupatorium odoratum* . Journal of Weed Science, 34(2), 7–11.

[ece311513-bib-0065] Zhu, G. , Li, H. , & Zhao, L. (2017). Incorporating anthropogenic variables into ecological niche modeling to predict areas of invasion of *Popillia japonica* . Journal of Pest Science, 90(1), 151–160. 10.1007/s10340-016-0780-5

