# Correction to “The Influence of Climate Change on the Potential Distribution of *Ageratum conyzoides* in China”

**DOI:** 10.1002/ece3.70895

**Published:** 2025-03-11

Wang, Y., Y. Yang, and M. Zhang. 2024. “The Influence of Climate Change on the Potential Distribution of Ageratum Conyzoides in China.” *Ecology and Evolution* **14**: e11513. 10.1002/ece3.11513.

In the last paragraph of the “Model establishment and evaluation” section, the text “Both measurements range from 0 to 1, with values closer to 1 indicating better model performance. AUC values ranging from 0.8 to 1 and TSS values ranging from 0.8 to 1 indicate high model reliability (Allouche et al. 2006; Eskildsen et al. 2013).” was incorrect. This should have read: “The AUC value ranges from 0 to 1, with higher values corresponding to greater predictive accuracy (Wang et al. 2007). The TSS value ranges from −1 to 1, with values closer to 1 indicating better model performance (Allouche et al. 2006; Eskildsen et al. 2013).”

Added references: Wang, Y., B. Xie, F. Wan, Q. Xiao, and L. Dai. 2007. “Application of ROC Curve Analysis in Evaluating the Performance of Alien species' Potential Distribution Models.” Biodiversity Science 15, no. 4: 365–372.

We apologize for this error.

