# Supplementary material for: The influence of climate change on the potential distribution of Ageratum conyzoides in China
Source: Ecol Evol. 2024 Oct 27;14(10):e11513. doi: 10.1002/ece3.11513 (PMC11512728; doi:10.1002/ece3.11513)
Supplement: Supplementary file 1 — Data S1. [file ECE3-14-e11513-s002.docx]

**The Influence of Climate Change on the Potential Distribution of *Ageratum conyzoides***

Supporting information

**TABLE S1.** Correlation analysis among retained eleven environmental variables

| **Environment Variable** | Slope | BIO10 | BIO11 | BIO13 | BIO14 | BIO3 | BIO7 | Aspect | T-TEB | T_SAND | T-pH-H_2_O |
| --- | --- | --- | --- | --- | --- | --- | --- | --- | --- | --- | --- |
| Slope | 1.000 |  |  |  |  |  |  |  |  |  |  |
| BIO10 | -.485 | 1.000 |  |  |  |  |  |  |  |  |  |
| BIO11 | -.300 | .475 | 1.000 |  |  |  |  |  |  |  |  |
| BIO13 | .122 | .220 | .046 | 1.000 |  |  |  |  |  |  |  |
| BIO14 | .103 | -.094 | .061 | 0.004 | 1.000 |  |  |  |  |  |  |
| BIO3 | .054 | -.325 | .597 | -.191 | .143 | 1.000 |  |  |  |  |  |
| BIO7 | -.112 | .182 | -.645 | .027 | -.357 | -.691 | 1.000 |  |  |  |  |
| Aspect | .049 | -0.019 | -.027 | .090 | -.029 | -.022 | -0.005 | 1.000 |  |  |  |
| T-TEB | 0.001 | .078 | .042 | -.054 | -0.004 | 0.011 | 0.020 | -0.009 | 1.000 |  |  |
| T_SAND | -.096 | .075 | .106 | -.094 | -.140 | .062 | -0.001 | -.030 | -.408 | 1.000 |  |
| T-pH-H_2_O | -.073 | .161 | .043 | -.092 | -.171 | -.080 | .117 | -.027 | .632 | -.059 | 1.000 |


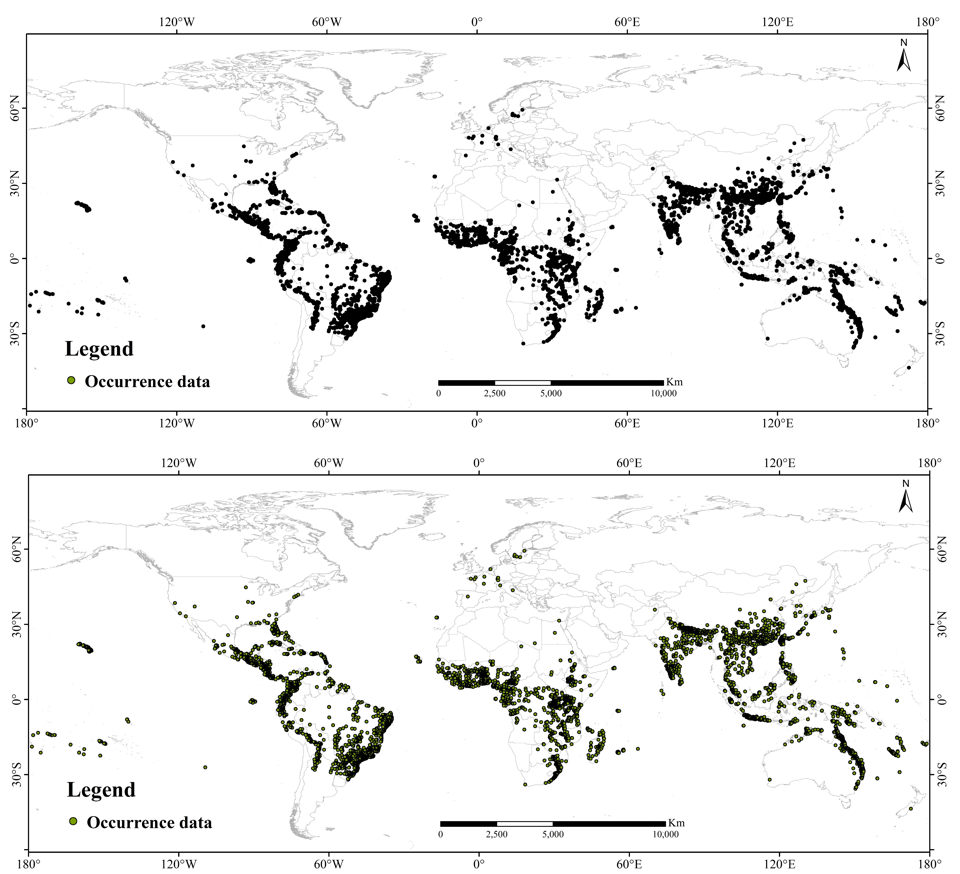


**Fig S1.** Global distribution site of *A. conyzoides* (The top is before filtering, and the bottom is after filtering).


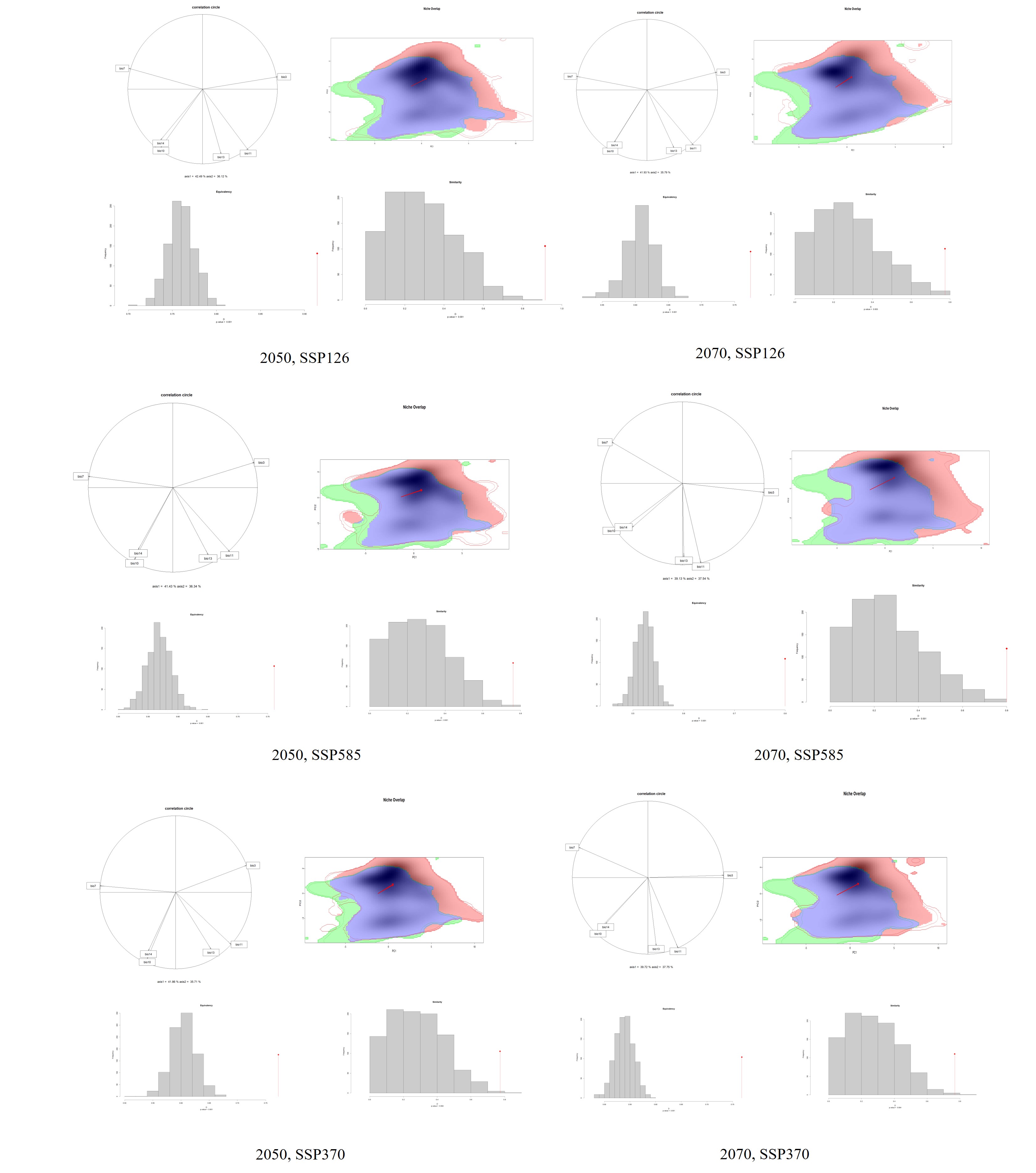


**Figure S2.** Climatic ecological niches of *A. conyzoides* under different climate scenarios. Picture of Correlation circle: Principal component analysis (PCA) on environmental variables. Picture of niche overlap: The purple, green and red shadows indicated niche overlap, unfilling, and expansion, respectively. The green soid and dashed lines represented 100% and 75% conditions of the background environment in present suitable distribution area, and the red soid and dashed lines represented 100% and 75% conditions of the background environment in future suitable distribution area, respectively. The solid and dashed arrows indicated the direction of niche transfer between the climate niche and the background environment from present and future suitable area. Picture of equivalence and similarity: Niche equivalence and similarity test between present and future suitable area.
