# Supplementary material for: The influence of climate change on the potential distribution of Ageratum conyzoides in China
Source: Ecol Evol. 2024 Oct 27;14(10):e11513. doi: 10.1002/ece3.11513 (PMC11512728; doi:10.1002/ece3.11513)
Supplement: Supplementary file 2 — Data S2. [file ECE3-14-e11513-s001.docx]

occs <- read.csv("huo.csv")

occs <- occs[,2:3]

colnames(occs) <- c("x", "y")

files=dir(pattern = "*.asc")

clim=list()

for (i in 1:length(files)) {

t_texture <- raster::stack(files[i])

clim[i] <- t_texture

}

bg <- dismo::randomPoints(clim[1], n = 30000) %>% as.data.frame()

result <- ENMevaluate(occs = occs[,1:2], envs = clim,

bg = bg[,1:2],

partitions = 'jackknife',

tune.args = list(fc=c("L", "LQ", "H", "LQH", "LQHP","LQHPT"),

rm = c(0.1, seq(0.5, 4, 0.5))),

algorithm = 'maxent.jar')

delta_AICc <- evalplot.stats(e = result,

stats = c("delta.AICc"),

color = "fc",

x.var = "rm",

error.bars = FALSE)

pdf('delta_AICc3.pdf', width = 5, height = 4)

print(delta_AICc3)

dev.off()

# Set working directory:

setwd("E:/R")

# ----------------------------------------------------------------------

bioclim<-

raster::stack(

c(

bio_3 ="D:/wc2.1_2.5m_bio/bio_3.tif",

bio_7 ="D:/wc2.1_2.5m_bio/bio_7.tif",

bio_10 ="D:/wc2.1_2.5m_bio/bio_10.tif",

bio_11 ="D:/wc2.1_2.5m_bio/bio_11.tif",

bio_14 ="D:/wc2.1_2.5m_bio/bio_14.tif",

bio_13 ="D:/wc2.1_2.5m_bio/bio_13.tif",

bio_Aspect ="D:/wc2.1_2.5m_bio/Aspect.tif",

bio_slope ="D:/wc2.1_2.5m_bio/slope.tif",

bio_TEB ="D:/wc2.1_2.5m_bio/TEB.tif",

bio_PH_H2O ="D:/wc2.1_2.5m_bio/T_PH_H2O.tif",

bio_SAND ="D:/wc2.1_2.5m_bio/SAND.tif"

))

plot(bioclim)

# ----------------------------------------------------------------------

huo<- read.csv("D:/huo.csv")

summary(huo)

# -------------------------------------------------------------------------

# Format Data with pseudo-absences : random method

myBiomodData <- BIOMOD_FormatingData (resp.var = huo_occ["suoluo"],

expl.var = bioclim,

resp.xy = huo_occ[, c("Longitude","Latitude")],

resp.name = "suoluo",

PA.nb.rep = 3,

PA.nb.absences = 30000,

PA.strategy = 'random',

filter.raster = TRUE

)

plot(myBiomodData)

# -------------------------------------------------------------------------

bm_DefaultModelingOptions()

myBiomodOptions <- BIOMOD_ModelingOptions(

MAXENT = list( linear = TRUE,

quadratic = TRUE,

product = TRUE,

threshold = FALSE,

hinge = TRUE,

betamultiplier = 2))

myBiomodOptions <-bm_DefaultModelingOptions()

# Model single models

myBiomodModelOut <- BIOMOD_Modeling(

weights = NULL,

bm.format = myBiomodData,

bm.options = myBiomodOptions,

modeling.id = 'AllModels',

models =c( "GLM", "GBM", "CTA", "FDA", "MARS", "RF", "MAXENT"),

CV.nb.rep = 10,

CV.perc = 0.75,

CV.strategy='random',

var.import = 3,

metric.eval = c("TSS", "ROC"))

# Represent evaluation scores

bm_PlotEvalMean(bm.out = myBiomodModelOut, dataset = 'calibration')

bm_PlotEvalMean(bm.out = myBiomodModelOut, dataset = 'validation')

bm_PlotEvalBoxplot(bm.out = myBiomodModelOut, group.by = c('algo', 'run'))

myBiomodModelOut

# Get evaluation scores & variables importance

get_evaluations(myBiomodModelOut)

get_variables_importance(myBiomodModelOut)

# Represent evaluation scores & variables importance

bm_PlotEvalMean(bm.out = myBiomodModelOut)

bm_PlotEvalMean(bm.out = myBiomodModelOut) %>%

.$tab %>%

DT::datatable()

bm_PlotEvalBoxplot(bm.out = myBiomodModelOut, group.by = c('algo', 'algo'))

# -------------------------------------------------------------------------

# Model ensemble models

myBiomodEM <- BIOMOD_EnsembleModeling(bm.mod = myBiomodModelOut,

models.chosen = "all",

em.by = "all",

em.algo = c( 'EMwmean'),

metric.select = "all",

metric.select.thresh = c(0.75,0.9),

metric.eval = c("TSS", "ROC"),

var.import = 3)

myBiomodEM

# Get evaluation scores & variables importance

get_evaluations(myBiomodEM)

get_variables_importance(myBiomodEM)

# Represent variables importance

try(varimp_em <- get_variables_importance(myBiomodEM))

try(write.csv(

x = varimp_em,

file = paste0("huoxiangji", "/", "huoxiangji", "_varimp_em_tab.csv")

))

# Represent response curves

try(rc1 <- bm_PlotResponseCurves(

bm.out = myBiomodEM,

models.chosen = get_built_models(myBiomodEM),

fixed.var = "median"

))

try(write.csv(

x = rc1$tab,

file = paste0("huoxiangji", "/", "huoxiangji", "_ResponseCurves_median_tab.csv")

))

try(rc1$plot)

try(ggsave(paste0(sp, "/", sp, "_ResponseCurves_median_plot.pdf")))

# Project single models

myBiomodProj <- BIOMOD_Projection(bm.mod = myBiomodModelOut,

proj.name = 'Current',

new.env =bioclim,

models.chosen ="all",

metric.binary = 'all',

metric.filter = 'all',

overwrite=TRUE)

#Project ensemble models

myBiomodEMProj <- BIOMOD_EnsembleForecasting(bm.em = myBiomodEM,

bm.proj = myBiomodProj,

proj.name = 'CurrentEM',

models.chosen ="all",

metric.binary = 'all',

metric.filter = 'all')

# Predicting the future

bioclim126_50<-

raster::stack(

c(

bio_3 ="D:/ worldclim/2050ssp126/bio_3.tif",

bio_7 ="D:/ worldclim/2050ssp126/bio_7.tif",

bio_10 ="D:/ worldclim/2050ssp126/bio_10.tif",

bio_11 ="D:/ worldclim/2050ssp126/bio_11.tif",

bio_14 ="D:/ worldclim/2050ssp126/bio_14.tif",

bio_13 ="D:/ worldclim/2050ssp126/bio_13.tif",

bio_Aspect ="D:/wc2.1_2.5m_bio/Aspect.tif",

bio_slope ="D:/wc2.1_2.5m_bio/slope.tif",

bio_TEB ="D:/wc2.1_2.5m_bio/TEB.tif",

bio_PH_H2O ="D:/wc2.1_2.5m_bio/T_PH_H2O.tif",

bio_SAND ="D:/wc2.1_2.5m_bio/SAND.tif"

))

# Project ensemble models (building single projections)

EMBCC126_50 <- BIOMOD_EnsembleForecasting(bm.em = myBiomodEM,

proj.name = 'EMBCC126_50',

new.env = bioclim126_50,

models.chosen = 'all',

metric.binary = 'all',

metric.filter = 'all',

overwrite=TRUE)

plot(EMBCC126_50)

# -------------------------------------------------------------------------

bioclim370_50<-

raster::stack(

c(

bio_3 ="D:/ worldclim2050ssp370/bio_3.tif",

bio_7 ="D:/ worldclim/2050ssp370/bio_7.tif",

bio_10 ="D:/ worldclim/2050ssp370/bio_10.tif",

bio_11 ="D:/ worldclim/2050ssp370/bio_11.tif",

bio_14 ="D:/ worldclim/2050ssp370/bio_14.tif",

bio_13 ="D:/ worldclim/2050ssp370/bio_13.tif",

bio_Aspect ="D:/wc2.1_2.5m_bio/Aspect.tif",

bio_slope ="D:/wc2.1_2.5m_bio/slope.tif",

bio_TEB ="D:/wc2.1_2.5m_bio/TEB.tif",

bio_PH_H2O ="D:/wc2.1_2.5m_bio/T_PH_H2O.tif",

bio_SAND ="D:/wc2.1_2.5m_bio/SAND.tif"

))

# Project ensemble models (building single projections)

EMBCC37_50 <- BIOMOD_EnsembleForecasting(bm.em = myBiomodEM,

proj.name = 'EMBCC370_50',

new.env = bioclim370_50,

models.chosen = 'all',

metric.binary = 'all',

metric.filter = 'all')

plot(EMBCC37_50)

# -------------------------------------------------------------------------

#BCC585_2050

bioclim585_50<-

raster::stack(

c(

bio_3 ="D:/ worldclim/2050ssp585/bio_3.tif",

bio_7 ="D:/ worldclim/2050ssp585/bio_7.tif",

bio_10 ="D:/ worldclim/2050ssp585/bio_10.tif",

bio_11 ="D:/ worldclim/2050ssp585/bio_11.tif",

bio_14 ="D:/ worldclim/2050ssp585/bio_14.tif",

bio_13 ="D:/ worldclim/2050ssp585/bio_13.tif",

bio_Aspect ="D:/wc2.1_2.5m_bio/Aspect.tif",

bio_slope ="D:/wc2.1_2.5m_bio/slope.tif",

bio_TEB ="D:/wc2.1_2.5m_bio/TEB.tif",

bio_PH_H2O ="D:/wc2.1_2.5m_bio/T_PH_H2O.tif",

bio_SAND ="D:/wc2.1_2.5m_bio/SAND.tif"

))

# Project ensemble models (building single projections)

EMBCC58_50 <- BIOMOD_EnsembleForecasting(bm.em = myBiomodEM,

proj.name = 'EMBCC585_50',

new.env = bioclim585_50,

models.chosen = 'all',

metric.binary = 'all',

metric.filter = 'all')

plot(EMBCC58_50)

# -------------------------------------------------------------------------

bioclim126_70<-

raster::stack(

c(

bio_3 ="D:/ worldclim/2070ssp126/bio_3.tif",

bio_7 ="D:/ worldclim/2070ssp126/bio_7.tif",

bio_10 ="D:/ worldclim/2070ssp126/bio_10.tif",

bio_11 ="D:/ worldclim/2070ssp126/bio_11.tif",

bio_14 ="D:/ worldclim/2070ssp126/bio_14.tif",

bio_13 ="D:/ worldclim/2070ssp126/bio_13.tif",

bio_Aspect ="D:/wc2.1_2.5m_bio/Aspect.tif",

bio_slope ="D:/wc2.1_2.5m_bio/slope.tif",

bio_TEB ="D:/wc2.1_2.5m_bio/TEB.tif",

bio_PH_H2O ="D:/wc2.1_2.5m_bio/T_PH_H2O.tif",

bio_SAND ="D:/wc2.1_2.5m_bio/SAND.tif"

))

# Project ensemble models (building single projections)

EMBCC126_70 <- BIOMOD_EnsembleForecasting(bm.em = myBiomodEM,

proj.name = 'EMBBC126_70',

new.env = bioclim126_70,

models.chosen = 'all',

metric.binary = 'all',

metric.filter = 'all')

plot(EMBCC126_70)

# -------------------------------------------------------------------------

bioclim370_70<-

raster::stack(

c(

bio_3 ="D:/ worldclim/2070ssp370/bio_3.tif",

bio_7 ="D:/ worldclim/2070ssp370/bio_7.tif",

bio_10 ="D:/ worldclim/2070ssp370/bio_10.tif",

bio_11 ="D:/ worldclim/2070ssp370/bio_11.tif",

bio_14 ="D:/ worldclim/2070ssp370/bio_14.tif",

bio_13 ="D:/ worldclim/2070ssp370/bio_13.tif",

bio_Aspect ="D:/wc2.1_2.5m_bio/Aspect.tif",

bio_slope ="D:/wc2.1_2.5m_bio/slope.tif",

bio_TEB ="D:/wc2.1_2.5m_bio/TEB.tif",

bio_PH_H2O ="D:/wc2.1_2.5m_bio/T_PH_H2O.tif",

bio_SAND ="D:/wc2.1_2.5m_bio/SAND.tif"

))

# Project ensemble models (building single projections)

EMBCC370_70 <- BIOMOD_EnsembleForecasting(bm.em = myBiomodEM,

proj.name = 'EMBBC370_70',

new.env = bioclim370_70,

models.chosen = 'all',

metric.binary = 'all',

metric.filter = 'all')

plot(EMBCC370_70)

# -------------------------------------------------------------------------

bioclim585_70<-

raster::stack(

c(

bio_3 ="D:/ worldclim/2070ssp585/bio_3.tif",

bio_7 ="D:/ worldclim/2070ssp585/bio_7.tif",

bio_10 ="D:/ worldclim/2070ssp585/bio_10.tif",

bio_11 ="D:/ worldclim/2070ssp585/bio_11.tif",

bio_14 ="D:/ worldclim/2070ssp585/bio_14.tif",

bio_13 ="D:/ worldclim/2070ssp585/bio_13.tif",

bio_Aspect ="D:/wc2.1_2.5m_bio/Aspect.tif",

bio_slope ="D:/wc2.1_2.5m_bio/slope.tif",

bio_TEB ="D:/wc2.1_2.5m_bio/TEB.tif",

bio_PH_H2O ="D:/wc2.1_2.5m_bio/T_PH_H2O.tif",

bio_SAND ="D:/wc2.1_2.5m_bio/SAND.tif"

))

# Project ensemble models (building single projections)

EMBCC585_70 <- BIOMOD_EnsembleForecasting(bm.em = myBiomodEM,

proj.name = 'EMBBC585_70',

new.env = bioclim585_70,

models.chosen = 'all',

metric.binary = 'all',

metric.filter = 'all')

plot(EMBCC585_70)

# -------------------------------------------------------------------------

nat<- read.csv("F:/current.csv")

inv<- read.csv("F:/future.csv")

pca.env<-dudi.pca(rbind(nat,inv)[,3:9],scannf=FALSE,nf=2)

ecospat.plot.contrib(contrib=pca.env$co, eigen=pca.env$eig)

scores.globclim<-pca.env$li # PCA scores for the whole study area

scores.sp.nat<-suprow(pca.env,nat[which(nat[,10]==1),3:12])$li

scores.sp.inv<-suprow(pca.env,inv[which(inv[,10]==1),3:12])$li

scores.clim.nat<-suprow(pca.env,nat[,3:9])$li

scores.clim.inv<-suprow(pca.env,inv[,3:9])$li

grid.clim.nat<-ecospat.grid.clim.dyn(glob=scores.globclim,

glob1=scores.clim.nat, sp=scores.sp.nat, R=1000, th.sp=0)

grid.clim.inv<-ecospat.grid.clim.dyn(glob=scores.globclim,

glob1=scores.clim.inv, sp=scores.sp.inv, R=1000, th.sp=0)

ecospat.niche.overlap (grid.clim.nat, grid.clim.inv, cor=TRUE)

eq.test<-ecospat.niche.equivalency.test(grid.clim.nat,

grid.clim.inv, rep=1000, ncores = 2)

sim.test<-ecospat.niche.similarity.test(grid.clim.nat,

grid.clim.inv, rep=1000, rand.type = 1,ncores = 2)

ecospat.plot.overlap.test(eq.test, "D", "Equivalency")

ecospat.plot.overlap.test(sim.test, "D", "Similarity")

ecospat.niche.dyn.index (grid.clim.nat, grid.clim.inv,

intersection=0.25)

ecospat.plot.niche.dyn(grid.clim.nat, grid.clim.inv, quant=0.25,

interest=2, title= "Niche Overlap", name.axis1="PC1",

name.axis2="PC2")

ecospat.shift.centroids(scores.sp.nat,scores.sp.inv, scores.clim.nat,scores.clim.inv)
